# The Shell of the Invasive Bivalve Species *Dreissena polymorpha*: Biochemical, Elemental and Textural Investigations

**DOI:** 10.1371/journal.pone.0154264

**Published:** 2016-05-23

**Authors:** Françoise Immel, Cédric Broussard, Bastien Catherinet, Laurent Plasseraud, Gérard Alcaraz, Irina Bundeleva, Frédéric Marin

**Affiliations:** 1 Laboratoire de Biogenèse Membranaire UMR5200, CNRS, Université de Bordeaux, Villenave d'Ornon, France; 2 Biogéosciences UMR6282, CNRS, Université de Bourgogne Franche-Comté, Dijon, France; 3 Institut Cochin, INSERM U1016, CNRS UMR8104, Université Paris Descartes, Paris, France; 4 Plate-forme Protéomique 3P5, Université Paris Descartes, Sorbonne Paris Cité, Paris, France; 5 ICMUB UMR6302, CNRS, Université de Bourgogne Franche-Comté, Dijon, France; 6 UPSP PROXISS, Département Agronomie Environnement AgroSupDijon, Dijon, France; University of Hong Kong, HONG KONG

## Abstract

The zebra mussel *Dreissena polymorpha* is a well-established invasive model organism. Although extensively used in environmental sciences, virtually nothing is known of the molecular process of its shell calcification. By describing the microstructure, geochemistry and biochemistry/proteomics of the shell, the present study aims at promoting this species as a model organism in biomineralization studies, in order to establish a bridge with ecotoxicology, while sketching evolutionary conclusions. The shell of *D*. *polymorpha* exhibits the classical crossed-lamellar/complex crossed lamellar combination found in several heterodont bivalves, in addition to an external thin layer, the characteristics of which differ from what was described in earlier publication. We show that the shell selectively concentrates some heavy metals, in particular uranium, which predisposes *D*. *polymorpha* to local bioremediation of this pollutant. We establish the biochemical signature of the shell matrix, demonstrating that it interacts with the *in vitro* precipitation of calcium carbonate and inhibits calcium carbonate crystal formation, but these two properties are not strongly expressed. This matrix, although overall weakly glycosylated, contains a set of putatively calcium-binding proteins and a set of acidic sulphated proteins. 2D-gels reveal more than fifty proteins, twenty of which we identify by MS-MS analysis. We tentatively link the shell protein profile of *D*. *polymorpha* and the peculiar recent evolution of this invasive species of Ponto-Caspian origin, which has spread all across Europe in the last three centuries.

## Introduction

In ecotoxicology, the usefulness of molluscs as sentinel organisms for tracing anthropic or natural pollution is well established [1]. Molluscs are indeed widespread and versatile and can be used in almost all environments including marine [2], freshwater [3], terrestrial [4] and in different manners, depending on their tolerance to pollutants. Some species like the freshwater pearl mussel *Margaritifera margaritifera*, which grows exclusively in pristine water, are unerring indicators of water purity [5], since they rapidly disappear when the environment is polluted, even very temporarily [6, 7]. Other species withstand pollution and exhibit the capacity to accumulate high content of heavy metals or organic pollutants in their living tissues and in their shell [8], without any apparent impairment of their physiological functions. An example of such shell accumulation is the edible mussel *Mytilus galloprovincialis*, whose shell nacreous layer is a faultless recorder of lead contaminations in Galizian Rias [9].

*Dreissena polymorpha* (sp.), also referred as the freshwater zebra mussel, can withstand significant environmental variations (‘euryocious’) [10]. This small bivalve is an invasive species that originates from the Ponto-Caspian area (the geographic zone covering the Caspian and Black Seas) [11–12] and that spread all across Europe via the waterways network, dug in the 18^th^-20^th^ centuries to link Eastern and Western Europe [13]. It was first used to monitor cadmium pollution in a lake near Hamburg (Germany) in 1985 [14] and, since then, has been largely employed to assess the contamination of freshwater systems by heavy metals [15], to monitor organic anthropogenic substances like polycyclic aromatic hydrocarbons (PAHs) [16] and, more recently, to investigate environmental effects of nanoparticles [17]. It is now recognized as a key-model in ecotoxicology and, because of its high filtration capacity [18], has become an accurate monitor for water quality management [19]. However, virtually nothing is known about the protein cortege used by this species for building its shell. This situation contrasts markedly to other model mollusc organisms, such as the pearl oyster [20–21], the giant limpet [22], the edible oyster [23], for which the use of molecular biology techniques [24] and of high throughput screening approaches [25–27] has recently allowed the identification of several shell proteins.

We assume that, similarly to the models cited above, *D*. *polymorpha* elaborates a composite shell made of calcium carbonate and of a minor organic fraction, according to a controlled biomineralization process [28]. For these models, it is known indeed that the secretion of the shell takes place at the interface between a specific organ, the mantle, and the growing shell itself, in a compartment sealed from outside by the periostracum, the organic layer that covers the outer part of the shell. In brief, the mantle extrudes the inorganic ionic precursors (mostly calcium and bicarbonate) together with a mixture of proteins, glycoproteins and polysaccharides, collectively called the shell matrix. All these ingredients react according to a self-assembling process, and the mineral formation occurs via a transient amorphous phase [29]. However, the successive steps of shell crystallites formation and packing are not yet elucidated.

The purpose of the present study is to establish, via a multi-approaches characterization, the basis for correlating, whenever possible, the microstructural, geochemical and biochemical signatures of the shell of *D*. *polymorpha* to the level of pollution of its surrounding environment. Such attempts have been already performed on other aquatic organisms: for example, when freshwater mussels, *Anodonta cygnea*, were incubated with heavy metals (Cd, Cu, Cr, Zn or Pb), Moura *et al*. [30] observed a decrease of protein, GAG and glucosamine concentrations in the extrapallial fluid of the animals exposed to lead, zinc and chromium, which suggested a reduction of the biomineralization process. For the purple sea urchin *Strongylocentrotus purpuratus*, several genes implicated in biomineralization like P19 and SM50 were up-regulated in lithium-treated and zinc-treated embryos [31].

In addition to linking biomineralization to ecotoxicology, our study emphasizes the use of the ordinary *Dreissena polymorpha* as a new and original model in biomineralization studies. In parallel, it represents, a preliminary attempt to correlate the proteomic shell signature of this invasive species to its very peculiar evolutionary and biogeographical history.

## Material and Methods

### Study area, shell sampling and ethics statement

The studied species is *Dreissena polymorpha* (Pallas), the zebra mussel, or wandering mussel. This species is a heterodont bivalve that belongs to the order Veneroida. It is a member of the small Dreissenidae family, which comprises only three extant genera, *Mytilopsis*, *Congeria* and *Dreissena* [32]. Morphologically, the shell of *D*. *polymorpha* exhibits a typical mytiliform shape (mussel shaped), flattened at anterior margin and ventrally, with an acute angle of the anterior part (<45°), while the posterior dorsal part is rounded. The outer part of the valves is characteristic with dark herringbone patterns (zebra) that are radially striped. The internal part of the valves is marked by the presence of a pallial line, rounded at posterior portion but with no sinus, and by the presence of two adductor muscle scars in posterior and anterior parts.

**Fig 1:**
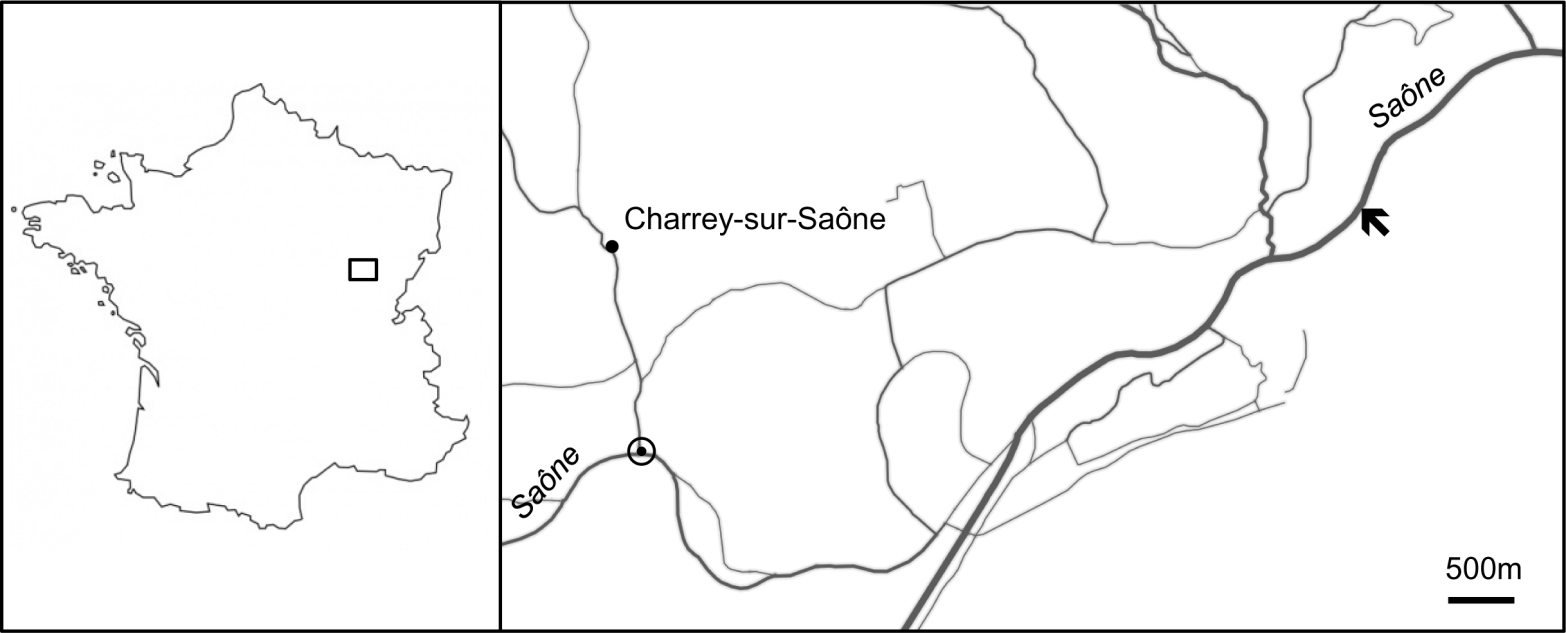
Location of the sampling site in the Saône River. Site is shown by the arrow (↖: 47° 08’ 82.5”N, 05° 24’ 08.5”E; ◉: 47° 07’ 36.4”N, 05° 16’ 58.4”E). Target (◉) indicates the water quality measuring station of Charrey-sur-Saône.

Specimens were sampled in the Saône river, at Charrey-sur-Saône (Côte d’Or, France), near a water monitoring station of the Rhône Méditerranée Corse Water Agency (DREAL Rhône-Alpes), as shown by Fig 1. Several hundred living zebra mussels (*Dreissena polymorpha*) were collected in October 2012 and held at 15°C in the laboratory in Saône waters before their dissection. The mussels were rapidly dissected, their soft tissues discarded and the shells cleaned overnight in dilute NaOCl (10% vol/vol, 0.26% active chlorine) at room temperature. Bleaching allowed removal of all superficial organic contaminants together with the outer organic layer, the periostracum. Shells were thoroughly rinsed twice with double distilled water before being air-dried.

*D*. *polymorpha* is an invasive species (not protected, not endangered). Specimens were sampled in accordance to an agreement delivered by the Préfecture de Côte d'Or to Biogeosciences for sampling wild animals in natural environments. The animals were sacrificed according to standard procedure.

### Shell mineralogy, elemental composition and microstructure

We used a total of 180 cleaned shells, which were powdered with an electrical mortar and pestle system (Pulverisette 2, Fritsch, Idar-Oberstein, Germany) and the resulting powder was sieved (200 μm) and homogenized. An aliquot of the shell powder was used for FT-IR characterization, in order to check the mineralogy of the shell (see below, § ‘Fourier Transform InfraRed spectroscopic characterization’ for further description).

Another aliquot was used for ICP-AES analysis. Minor and trace elements were determined by FILAB (Dijon, France), according to an internal procedure. In brief, one gram of homogenized cleaned powder was dissolved in 50 mL an aqueous solution containing in excess 3 mL of nitric acid and 9 mL of chlorhydric acid, and the solution was heated for ensuring complete dissolution. The solution was analysed on a ICP AES PERKIN 7300 system. In addition, the level of calcium was determined by flame atomic absorption spectrometry (AAS 3300, AAnalyst 400 Perkin–Elmer, Rodgau, Germany) with an uncertainty of ±2% and a detection limit of 0.5 μM. In parallel, the physicochemical characteristics of the water column close to the shell sampling location was obtained by DREAL Rhône-Alpes, as well as its heavy metal and metalloid contaminant content. Similarly, the content of the sediments in inorganic ions was also provided.

For microstructural investigation via scanning electron microscopy (SEM) observations, two shell valves (right or left) were included in Epoxy resin and the blocks were sliced (200 μm) with a saw microtome (Leica Biosystems, Newcastle, UK). Sections were parallel and perpendicular to the opening plan of the valves. The slices were then UV-glued on glass plates and polished with alumina suspension (0.05 μm, ESCIL, Chassier, France). The preparations were subsequent rinsed in an ultrasound bath, briefly etched with EDTA (1% wt/vol, 2min), rinsed with water and dried. Shell microstructures were observed with a tabletop scanning electron microscopy (Hitachi TM1000) and with a Field Emission Scanning Electron Microscope (FE-SEM, JSM-7600 F). In this latter case, the preparations were carbon-coated (10 nm thick) with a Cressington 308R Desktop Advanced Coating System. A set of additional freshly fractured shells was directly observed with the tabletop SEM, without any specific preparation.

### Matrix extraction

The shell powder was re-suspended in 10mL of distilled water and decalcified overnight by adding progressively cold dilute acetic acid (10% vol/vol) at a flow rate of 0.1 mL every 5 sec, according to our standardized procedure [33]. The final pH was 4.2. The clear solution was then centrifuged (3900 G, 30 min.). The supernatant containing the acid soluble matrix (ASM) was then filtered (5 μm) before being ultra-filtered for volume reduction on an Amicon cell (400 mL; cutoff 10kDa); the solution (10–15 mL) was then extensively dialyzed against Milli-Q water for three days (6 water changes) and lyophilized. The pellet containing the acid insoluble matrix (referred as AIM) was rinsed several times with Milli-Q water and lyophilized. In total, three batches of extracts (both AIM and ASM) were obtained. All the lyophilisates were weighed on a precision balance (Precisa XT120A model) three to five times, and the mean value was calculated.

### Fourier Transform InfraRed spectroscopic characterization (FT-IR)

FT-IR spectroscopy was used for checking the shell mineralogy of *Dreissena polymorpha*, as well as the overall chemical properties of the extracted matrices (ASM and AIM): to this end, in both cases, minute amounts of samples (shell powder or freeze-dried chips (< 1 mg) of ASM and of AIM) were analyzed with a Bruker Vector 22 instrument (Bruker Optics Sarl, Marne la Vallée, France) fitted with a Specac MKII Golden Gate Diamond Attenuated Total reflectance (ATR) device equipped with ZnSe lenses (Specac Ltd, Orpington, UK) in the 4000–500 cm^-1^ wavenumber range (twelve scans at a spectral resolution of 4 cm^-1^). The qualitative assignment of absorption bands was performed by comparison with previous spectra descriptions, achieved by our group or available in the bibliography [34, 35].

### Monosaccharide analysis

The monosaccharide content of AIM and ASM was obtained by suspension and homogenization of these lyophilisates in 2 M trifluoroacetic acid and subsequent hydrolysis at 105°C for 4 h under nitrogen atmosphere. This hydrolytic procedure allows releasing most monosaccharides (neutral, aminated, acidic) from complex mixtures (without degrading them), except sialic acids, which are destroyed, and the acetylated forms of glucosamine and galactosamine, which are converted to their respective non-acetylated forms. Samples were then evaporated to dryness before being dissolved in 100 μL of 20 mM NaOH. The neutral, amino and acidic sugar contents of hydrolysates were determined by High-Performance Anion-Exchange chromatography with Pulsed-Amperometric-Detection (HPAE-PAD) on a CarboPac PA 100 column (Dionex Corp., Sunnyvale, CA, USA). As blank controls, non-hydrolysed ASMs and AIMs were analysed, in order to detect potential free monosaccharides that may distort the results and lead to an over-representation of some sugar residues.

### Enzyme-Linked Lectin Assay (ELLA)

The zebra mussel ASM was screened for its lectin-binding profile via Enzyme-Linked Lectin Assay (ELLA) [36]. To this end, a set of 21 biotinylated lectins was used. Binding preferences and specificities of each lectin were checked by a thorough survey of different bibliographical sources [37–48]. The complete information on the binding specificities is summarized in the S1 Table.

The test is performed in 96-well plates (MaxiSorp, Nunc^TM^, Roskilde, Denmark), as previously described [49]: the zebra mussel ASM (50 ng/well) was incubated for 90 min at 37°C, followed by three washing steps with TBS/Tween20. The wells were then blocked with 1X concentrated Carbo-free blocking solution (Vector Laboratories, Orton Southgate, Peterborough, UK; ref. SP-5040) for 60 min at 37°C. The lectins (Vector Laboratories, Orton Southgate, Peterborough, UK; ref. BK-1000, -2000, -3000) were all diluted 1:100 from the stock solutions (2 mg/mL) with TBS/Tween20 and incubated into the microplate for 90 min at 37°C. After three washes with TBS/Tween20, Avidin-Alkaline Phosphatase (Avidin-AP, Sigma A7294, St. Louis, MO, USA) was used in a dilution of 1:70000 for 90 min at 37°C for the detection of bound biotinylated lectins. The wells were then washed five times with TBS/Tween20. ELISA substrate solution (aqueous diethanolamine, 9.7% vol/vol, pH adjusted to 9.8 with HCl) containing Phosphatase Substrate (0.5 mg/mL, pNPP Tablet, Sigma, St. Louis, MO, USA) was added to the wells (100 μL/well) and incubated at 37°C. The microplate was read at 405 nm every 15 min (BioRad Model 680). Different control experiments were included in the test: check of the background signal without ASM, lectin or Avidin-AP; negative control with ASM and lectin, but without Avidin-AP; negative control with ASM, without lectin, but with Avidin-AP. None of the negative controls showed a reaction with the substrate solution. The test was performed with triplicates of each lectin. The results were normalized and translated in percentage of reactivity by subtracting the negative control (ASM without lectin but with Avidin-AP) of all values and considering the highest response (Wheat germ agglutinin, WGA) as 100%. The standard deviation was calculated from the variation of the triplicate measurements. The test was performed twice.

### *In vitro* functional assays: inhibition and crystallization

The effect of the zebra mussel ASM on calcium carbonate was tested according to two procedures: inhibition and interference (crystallization) assays. In the first case, the ASM was checked for its capacity to inhibit the *in vitro* precipitation of calcium carbonate [50]. Two mL of 20 mM CaCl_2_ were rapidly added to 2 mL of 20 mM NaHCO_3_ containing variable amounts of protein extract (10, 20 and 30 μg). For each experiment, the pH was recorded with a mini glass electrode (6mm, Mettler-Toledo) coupled to a computer-connected pH-meter (Crison, GLP21). The pH was measured in continuous during 800 sec. Each ASM concentration was tested in duplicate. Between each experiment with a shell matrix aliquot, the electrode was refreshed with 0.5 M HCl, and negative controls were performed in the absence of protein, to check the stability of the electrode response.

In the second case, *i*.*e*., interference (crystallization) assay, the ASM was tested for its capacity to interfere with the growth of calcium carbonate crystals. The test was derived from that published by Albeck and coworkers [51]. In brief, calcite crystals were grown by slow diffusion of ammonium bicarbonate vapours into a CaCl_2_ solution (200 μL). The following modification was applied: the 10 mM CaCl_2_ solutions containing different quantities of ASM (serial dilutions from 25 μg/mL to 0.39 μg/mL) were incubated in 16-well culture slides (Lab-Tek, Nunc^TM^, Roskilde, Denmark). The slides were closed with their plastic cover, and sealed with parafilm. Prior to the experiments, the cover was pierced (1 mm diameter holes) in the middle of each well, to allow diffusion of the vapours only through the holes. The slides were placed in a 5 L closed desiccator containing crystals of ammonium bicarbonate, and incubated at 4°C for 48 h. Blank tests (without any matrix) were performed in a similar manner. The solution was then gently removed using a blunt-ended syringe needle connected to a vacuum pump, and the culture slide was incubated further at 37°C. The glass slides were mechanically dissociated from the plastic wells and directly observed (without carbon sputtering) with a tabletop SEM (Hitachi TM1000). The assay was repeated four times.

### Gel electrophoresis

#### Mono-dimensional gels

The shell extracts were analysed by conventional mono-dimensional SDS-PAGE (Bio-Rad, Mini Protean III gels), on precast gradient gels (Mini-PROTEAN TGX Gel 4–20% acrylamide, 90 mm x 70 mm, BioRad). Prior migration, the samples were treated as follows: lyophilisates of the ASM were dissolved in 2X Laemmli Sample Buffer (LSB) to a final matrix concentration of 5 μg/μL. One lyophilisate of the AIM was suspended in 2X LSB (5 mg in 500 μL). Both preparations were heat-denatured at 100°C: 5 min. for the ASM and 10 min. for the AIM. The LSB-solubilized fraction of the AIM is referred as LS-AIM (Laemmli soluble, acetic acid insoluble). The preparations were cooled down and gently centrifuged before being applied on the top of the gel: 20 μg of ASM and 50 μg of LS-AIM were applied, respectively. After migration, the gels were stained with Instant Blue Coomassie (Expedeon, Harston, UK), with silver nitrate [52], with Alcian Blue at low pH (pH 1) [33–53] and with ‘Stains-all’ [54]. While silver nitrate is supposed to stain most of the macromolecular components of the shell, Alcian Blue in acidic conditions stains mostly sulphate groups of polyanionic polysaccharides. ‘Stains-all’ stains blue the putative calcium-binding proteins, while leaving the non-calcium-binding proteins red or pink.

#### Bi-dimensional gels

ASM and AIM lyophilisates were prepared according to the manufacturer’s instructions. The samples were migrated on a 2D-gel Protean IEF cell (Bio-Rad), in the first dimension on strips before being fractionated on the second dimension on 4–20% gels. More precisely, a 7 cm linear pH 3–10 immobilized pH gradient IPG strip (ReadyStrip, BioRad) was re-hydrated overnight with 200 μg of protein sample in rehydration buffer (8M urea, 2M thiourea, 2% w/v CHAPS, 10mM DTT, 0.5% v/v pH 3–10 IPG buffer, 0.6% DeStreak reagent) and IEF was processed at 20°C (250 V for one hour, then a gradient voltage was applied to reach 4000 V in 3 hours followed by a constant step at 4000 V until 10,000 Vh). The strip was transferred successively in each two equilibration buffers, before being rinsed in TGS buffer (25mM Tris-HCl pH8.3, 192 mM glycine 0.1% SDS), positioned on top of precast gradient gel (Mini-PROTEAN TGX Gel 4–20% acrylamide, 90 mm x 70 mm, BioRad), and fixed in place with an overlay solution of 0.5% agarose **⁄**TGS (w/v). The ASM gel was stained with Instant Blue Coomassie. Because of the low amount of materials obtained from the AIM, the gel was stained with silver nitrate [52].

### MS/MS

MS/MS analyses were conducted on the two unfractionated bulk matrices, ASM and AIM, which were digested in-solution and in-gel, after a short migration in acrylamide gel. In-gel digestions were carried out with trypsin according to a published procedure with minor adjustments [55–56]: samples were destained twice with a mixture of 100 mM ammonium bicarbonate (ABC) and 50% (v/v) acetonitrile (ACN) for 45 min at 22°C and then dehydrated using 100% ACN for 15 min, before being reduced with 25 mM ABC containing 10 mM DTT for 1 h at 60°C and alkylated with 55 mM iodoacetamide in 25 mM ABC for 30 min in the dark at 22°C. Gel pieces were washed twice with 25 mM ABC and dehydrated (twice, 15 min) and dried (10 min) with 100% ACN. Gel cubes were incubated with sequencing grade modified trypsin (Promega, USA; 12.5 ng/μl in 40 mM ABC with 10% ACN, pH 8.0) overnight at 37°C. After digestion, peptides were washed with 25 mM ABC, dehydrated with 100% ACN and extracted twice with a mixture of 50% ACN–5% formic acid (FA). Extracts were dried using a vacuum centrifuge Concentrator plus (Eppendorf).

For in-solution digestion, the protocol was the same as in-gel digestion, with minor differences: no destain, no washes and no extraction but with reduction, alkylation and digestion with sequencing grade modified trypsin (Promega, USA; 0.1 μg/μl in 40 mM ABC with 10% ACN, pH 8.0). Extracts were dried as above and peptides were cleaned with ziptips C_18_ (Millipore) before MS analysis.

For MS and MS/MS ORBITRAP, analyses were performed using an Ultimate 3000 Rapid Separation Liquid Chromatographic (RSLC) system (Thermo Fisher Scientific) online with a hybrid LTQ-Orbitrap-Velos mass spectrometer (Thermo Fisher Scientific). Briefly, peptides were dissolved in 4 μL of 10% ACN-0.1% FA. Peptides were loaded and washed on a C_18_ reverse phase pre-column (3 μm particle size, 100 Å pore size, 150 μm i.d., 1 cm length). The loading buffer contained 98% H_2_O, 2% ACN and 0.1% TFA. Peptides were then separated on a C_18_ reverse phase resin (2 μm particle size, 100 Å pore size, 75 μm i.d., 15 cm length) with a 1 hour gradient from 100% A (0.1% FA and 100% H_2_O) to 50% B (80% ACN, 0.085% FA and 20% H_2_O).

The Linear Trap Quadrupole Orbitrap mass spectrometer acquired data throughout the elution process and operated in a data dependent scheme with full MS scans acquired with the Orbitrap, followed by up to 20 LTQ MS/MS CID spectra on the most abundant ions detected in the MS scan. Mass spectrometer settings were: full MS (AGC: 1*10^6^, resolution: 6*10^4^, m/z range 400–2000, maximum ion injection time: 500 ms); MS/MS (AGC: 5*10^3^, maximum injection time: 20 ms, minimum signal threshold: 500, isolation width: 2 Da, dynamic exclusion time setting: 30 s). The fragmentation was permitted of precursor with a charge state of 2, 3, 4 and up. For the spectral processing, the software used to generate.mgf files is Proteome discoverer 1.3. The threshold of Signal to Noise for extraction values is 3.

Database searches were carried out using Mascot version 2.4 and 2.5 (MatrixScience, London, UK) on ‘Other Metazoa’ proteins (2,282,777 sequences) and on ‘Other Eukaryota’ proteins (1,030,911 sequences) from NCBInr databank containing 67,337,701 residues (May 2015) (www.ncbi.nlm.nih.gov/). A fusion database merging a homemade shell proteins databank (762 sequences containing 220545 residues) and three additional databases (*Dreissena* Expressed Sequence Tags (ESTs) extracted from NCBI; the translated *Bathymodiolus azoricus*, *Pecten maximus* and *Laternula elliptica* transcriptomes) described in an earlier publication [57] was also checked. Two specific proteins databank—one from the sea urchin *Paracentrotus lividus* with restricted access (see http://octopus.obs-vlfr.fr/), constructed and provided by Laboratoire de Biologie du Développement, UMR CNRS 7009, Villefranche sur Mer–and the second one–from the genus *Crassostrea* (62,050 sequences)–were also included in the *in silico* analysis. The search parameters were as follows: carbamidomethylation as a variable modification for cysteins and oxidation as a variable modification for methionines. Up to 3 missed tryptic cleavages were tolerated and mass accuracy tolerance of 10 ppm for precursors and 0.45 Da for fragments were used for all tryptic mass searches. The protein score should be above 50 for ‘Other Metazoa’ and ‘Other Eukaryota’ databases and 40 for the other databases while the individual ions score was above 20 for all databases.

## Results

### Mineralogy and chemistry of the shell of *Dreissena polymorpha*

**Fig 2:**
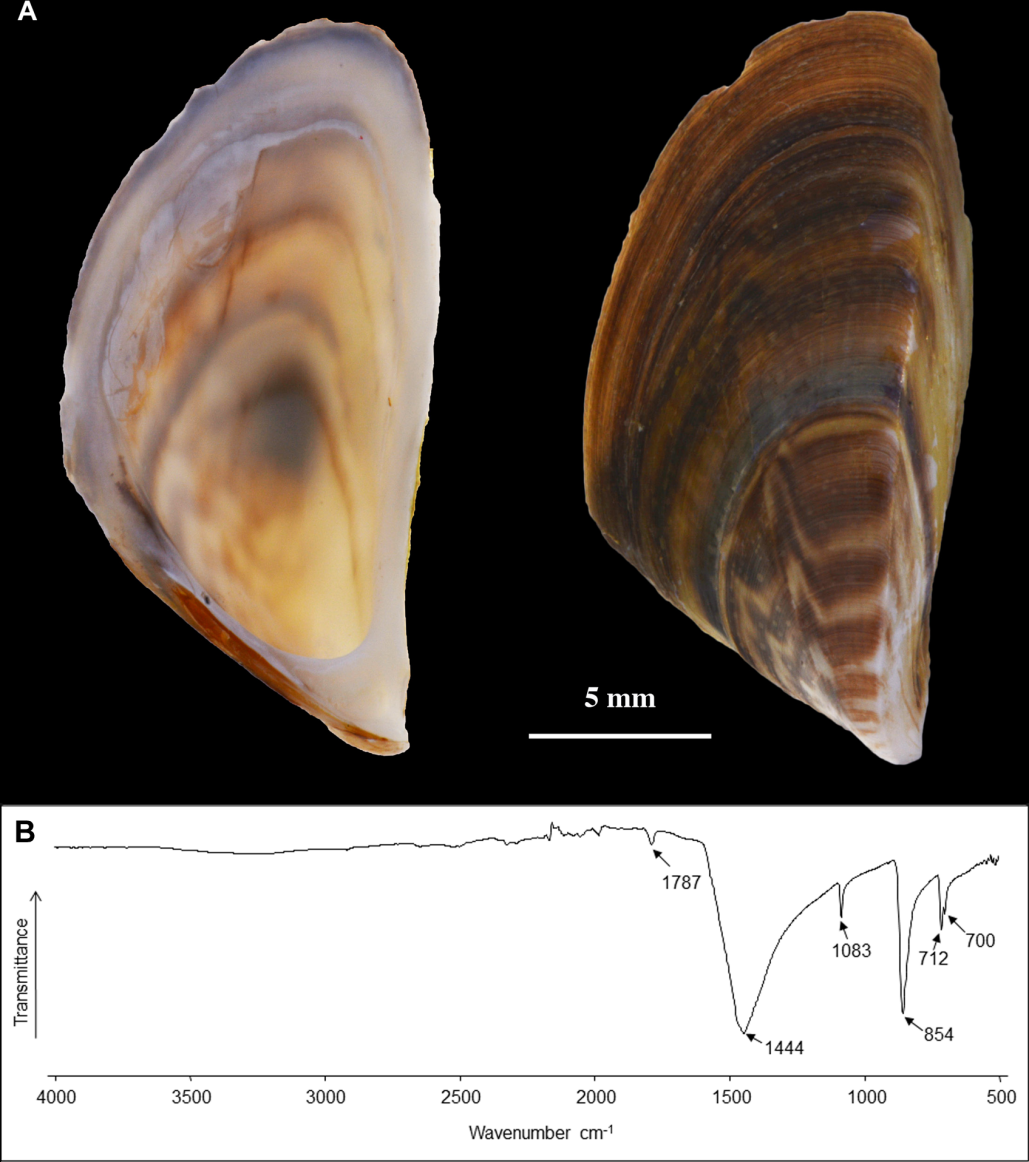
A) Inner and dorsal view of the *Dreissena polymorpha* shell. B) Infrared spectra of the shell powder.

We verified that the shell of *Dreissena polymorpha* is fully aragonitic (Fig 2B). The typical absorption bands of aragonite type structure are highlighted by the four characteristic vibration modes of CO_3_^2-^: *ν*_3_(1445 cm^-1^), *ν*_1_(1082 cm^-1^), *ν*_2_(854 cm^-1^) and *ν*_4_(712–699 cm^-1^) [58].

Tables 1, 2 and 3 show the elemental composition of the shell, the water column and the sediments. Most of the elements are present in low concentrations in the shell: except Ba (53 ppm), Sr (382 ppm) and U (14 ppm) (Table 2). Some elements are depleted in the shell but concentrated in the sediments (B, As, Co, Cu, Mn, Ti, Ni and Pb) with the exception of uranium (Table 2), which is concentrated in the shell relative to the sediment with an accumulation factor of 35879 (Table 3). The most noticeable data is the concentration factor of uranium in the shell.

**Table 1:** Chemistry of the water and the shell of *Dreissena polymorpha*.

	Temp.	Cond.	pH	TH	O_2_	COD	DBO	C	Ca	Cl	CO_3_	HCO_3_
	°C	μS cm^-1^		°f	(mg L^-1^)							
Water	12.2	484	8.7	20.7	9.2	16	0.6	4.5	73.7	33.5	0	187
Shell (mg kgDW^-1^)								10.03%	35%	71	nd	nd
	K	Mg	N	Na	NH_4_	NO_3_	NO_2_	P	PO_4_	SiO_2_	SO_4_	S
Water	3.3	5.47	<1	15.7	<0.05	17.7	0.07	0.12	0.23	7.3	25.4	nd
Shell (mg kgDW^-1^)	10	15	nd	2297	nd	nd	nd	3.9	nd	nd	nd	82

Water chemistry has been evaluated in October 2012 by the Rhône Méditerranée Corse Water Agency (DREAL Rhône-Alpes). nd: not detected.

**Table 2:** Contaminant concentrations in the water column, the sediments of the Saône River at the measuring station of Charrey-sur-Saône and the shell powder.

	Metals												
	Al	Ag	As	B	Ba	Be	Cd	Co	Cr	Cu	Fe	Hg	Mn
Water column (μg L^-1^)	nd	<0.02	1.5	15	41.1	0.01	<0.03	0.26	<0.5	1.4	nd	<0.02	nd
Sediments (mg kgDW^-1^)	35800	0.3	17.7	27.8	496	1.9	0.3	8.9	47.9	18.9	18900	0.084	385
Shell (mg kgDW^-1^)	<1	1.5	<1	<1	53	<1	<1	<1	<1	<1	2.2	<0.05	4.9
	Mo	Ni	Pb	Sb	Se	Sn	Sr	Te	Ti	Tl	U	V	Zn
Water column (μg L^-1^)	<1	<0.5	0.15	<0.5	<0.3	<0.5	nd	<0.5	6.1	<0.03	0.39	1	2
Sediments (mg kgDW^-1^)	0.5	20.5	26	1.2	1.3	3.9	nd	<0.2	1710	1.1	2	56.5	99.5
Shell (mg kgDW^-1^)	<1	<1	<1	<1	<1	<1	382	<1	<1	<1	14	<1	1.6

Contaminant concentrations in water column and sediments have been evaluated in October 2012 by the Rhône Méditerranée Corse Water Agency (DREAL Rhône-Alpes). nd: not detected.

**Table 3:** Accumulation factor and distribution coefficient of the most significant elements found in the shell and the water.

	Metals and elements							
	Ba	U	Zn	Cl	K	Mg	Na	P
Accumulation Factor (Me_Shell_/Me_Water_)	1290	35897	800	2	3	3	146	33
D = Distribution coefficient (Me/Ca)_Shell_/(Me/Ca)_Water_	0.2715	7.5590	0.1685	0.0004	0.0006	0.0006	0.0308	0.0068

### Microstructure

**Fig 3:**
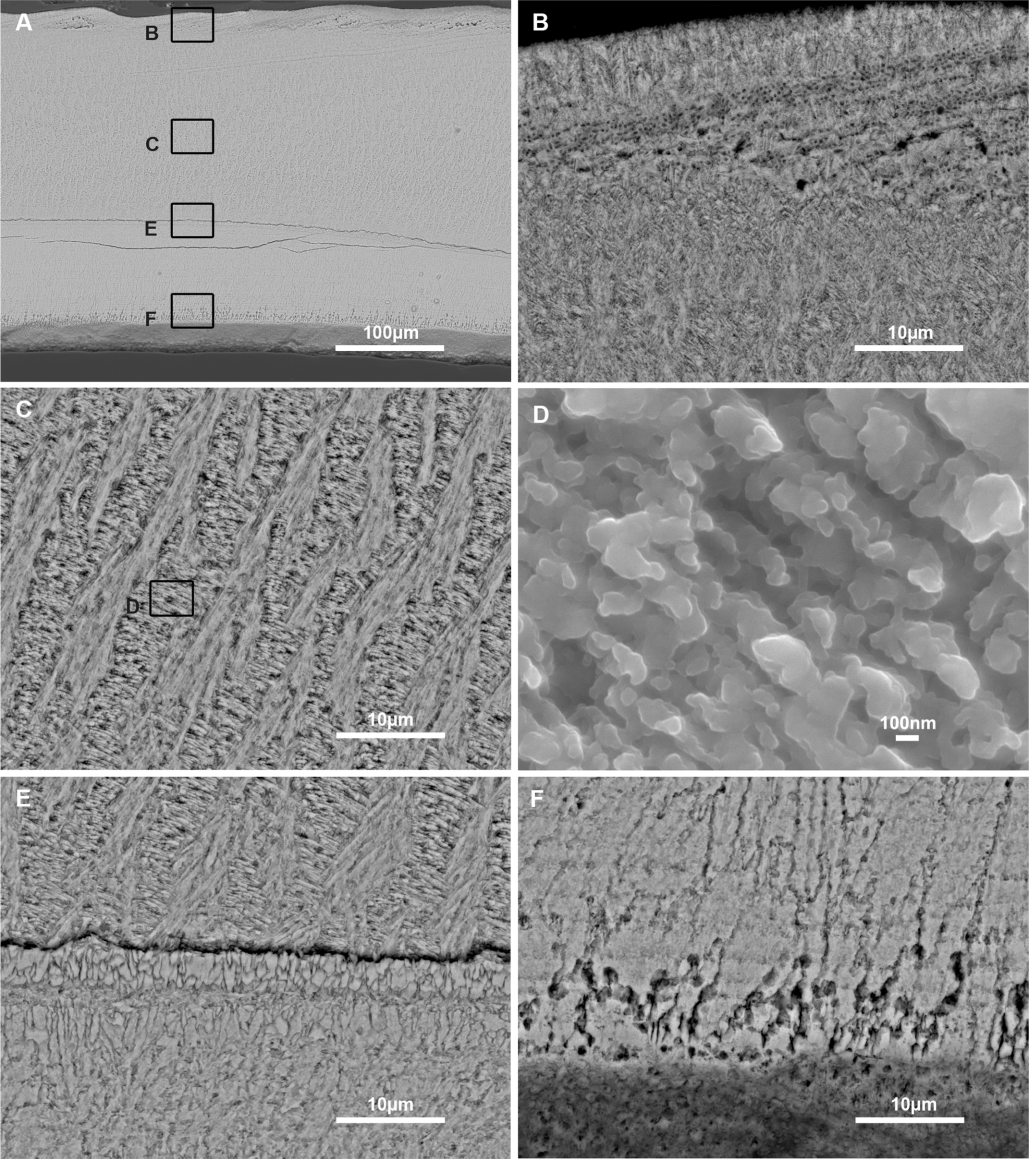
Secondary electron images of the shell microstructure of *Dreissena polymorpha*. Black boxes show location of the other views. A) Transversal section. B) Thin uppermost layer constitutes chevron-like columns. C) Crossed-lamellar layer. D) Crossed-lamellar fibres at high magnification. E) Crossed-lamellar layer based on a prismatic aggregate myostracal layer. The lower layer of the view is a complex crossed-lamellar layer. F) Innermost layer of the shell.

Fig 3 depicts the different microstructures observed in transverse section throughout the thickness of the shell (Fig 3A). The description below goes from top (outer) to bottom (internal). The uppermost layer is thin (< 10 μm, Fig 3B) and constitutes columnar chevron-like (*i*.*e*., ‘herringbone’) patterns, each column, of about 3–5 μm thick, being developed perpendicularly to the outer surface. This layer corresponds to the simple lamellar structure described by Archambault-Guezou [59]. Below this layer, one finds a zone of irregularly distributed pores (diameter about 200–300 nm). This transition layer exhibits a variable thickness: from a few micrometres (Fig 3B, left part) to more than 15 μm (Fig 3B, right part). In the larger part, pores are organized in successive plans (three or more), between which there are either sub-chevron patterns or spherulites. Below this transition layer, the main layer has a typical crossed-lamellar structure, characterized by alternating fibre bundles developed in two different plans (Fig 3C). At high magnification, each fibre (diameter about 200–300 nm), when slightly etched with EDTA, constitutes alignments of submicrometric granules (size from 100 to 300 nm, Fig 3D). The predominant crossed-lamellar layer is separated from the subjacent layer by a zone of lesser resistance, (marked by a fracture, Fig 3E), where the microstructure changes: this zone—4–5 μm thick—is characterized by irregular block-like rectangular to fusiform crystals. It is described by Archambault-Guezou as the prismatic aggregate myostracal layer with a fibrous fracture pattern [59]. The underlying layer is the so-called complex crossed-lamellar, according to the description of Taylor et al. [60]. It is composed of thin fibres reminiscent of the crossed-lamellar layer, although the absence of general emerging patterns and the multiple orientations of the fibres make it more difficult to describe (Fig 3E). The basal part of this layer is characterized by the abundance of pores that develop in the last ten μm from the internal surface of the shell (Fig 3F).

### Matrix extraction and FT-IR spectroscopy analysis

Table 4 shows the amount of organic matrix extracted from the shell powder, after decalcification. The ASM represents about 0.032–0.033% of the shell powder weight, while the AIM is about 0.040–0.043%. This represents an ASM/AIM ratio close to 1.

**Table 4:** Quantification of the ASM and AIM organic matrix from *Dreissena polymorpha* shell.

Powder	25g	% of matrix
Acid soluble matrix (ASM)	8.0–8.4 mg	0.032–0.033%
Acid insoluble matrix (AIM)	10.2–10.8 mg	0.040–0.043%
Total	18.2–19.2 mg	0.072–0.076%

**Fig 4:**
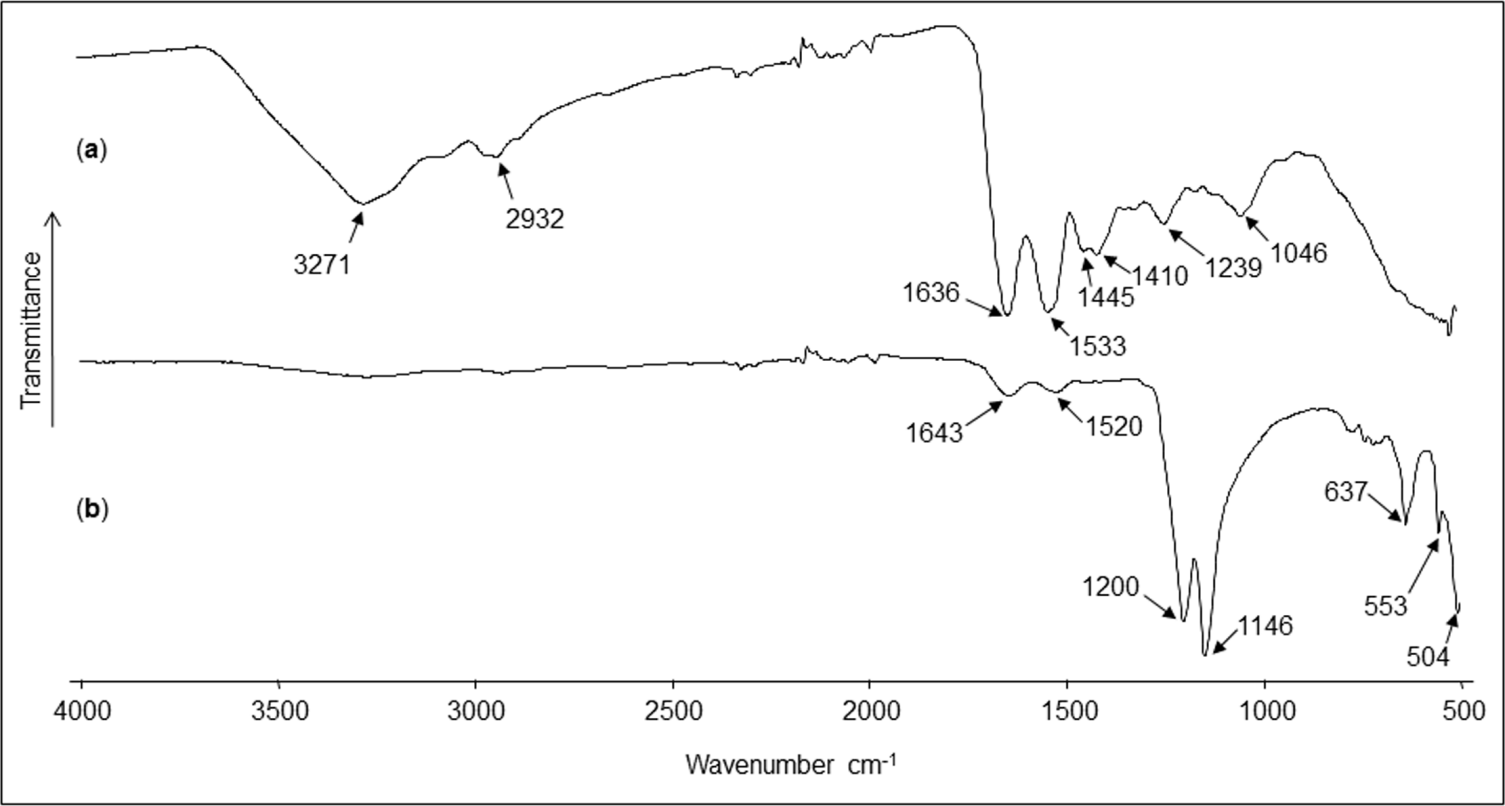
Infrared spectra of the acid soluble and the acid insoluble matrices. a) ASM and b) AIM.

The FT-IR (ATR) profiles of the ASM and AIM dry lyophilized fractions are shown in Fig 4A and 4B, respectively (transmittance mode). Regarding the ASM fraction, the spectrum reveals the characteristic vibrational bands of glycoproteins [49,61]. Thus, the broad band located at 3271 cm^-1^ was attributed to the amide A group (*ν*_N‒H_), the weaker bands centered at 2932 cm^-1^ were assigned to the C‒H stretching vibrations, and the absorptions at 1636 and 1533 cm^-1^ were ascribed to the amide I (*ν*_C = O_) and II (*ν*_C‒N_) bands, respectively. The absorptions at 1445 and 1410 cm^-1^ are in accordance with asymmetric C‒H scissoring vibrations (*δ*_as_), while the one at 1239 cm^-1^ can be linked to the amide III band (*ν*_C‒N_/*δ*_N‒H_). The polysaccharide composition of the fraction is evidenced by the broad absorption at 1046 cm^-1^ assigned to C‒O stretching vibrations. The AIM spectrum is simpler, showing mainly two meaningful groups of bands located in the ranges of 1200–1100 and 650–500 cm^-1^ that can be reasonably attributed to P‒O stretching and O‒P‒O bending vibrations, respectively, and matching with the presence of phosphates (PO_4_^3-^) [62]. The amide I and amide II bands are still visible at 1643 and 1520 cm^-1^, but strongly reduced in their amplitude.

### Monosaccharide composition and lectin profiling of the ASM

**Fig 5:**
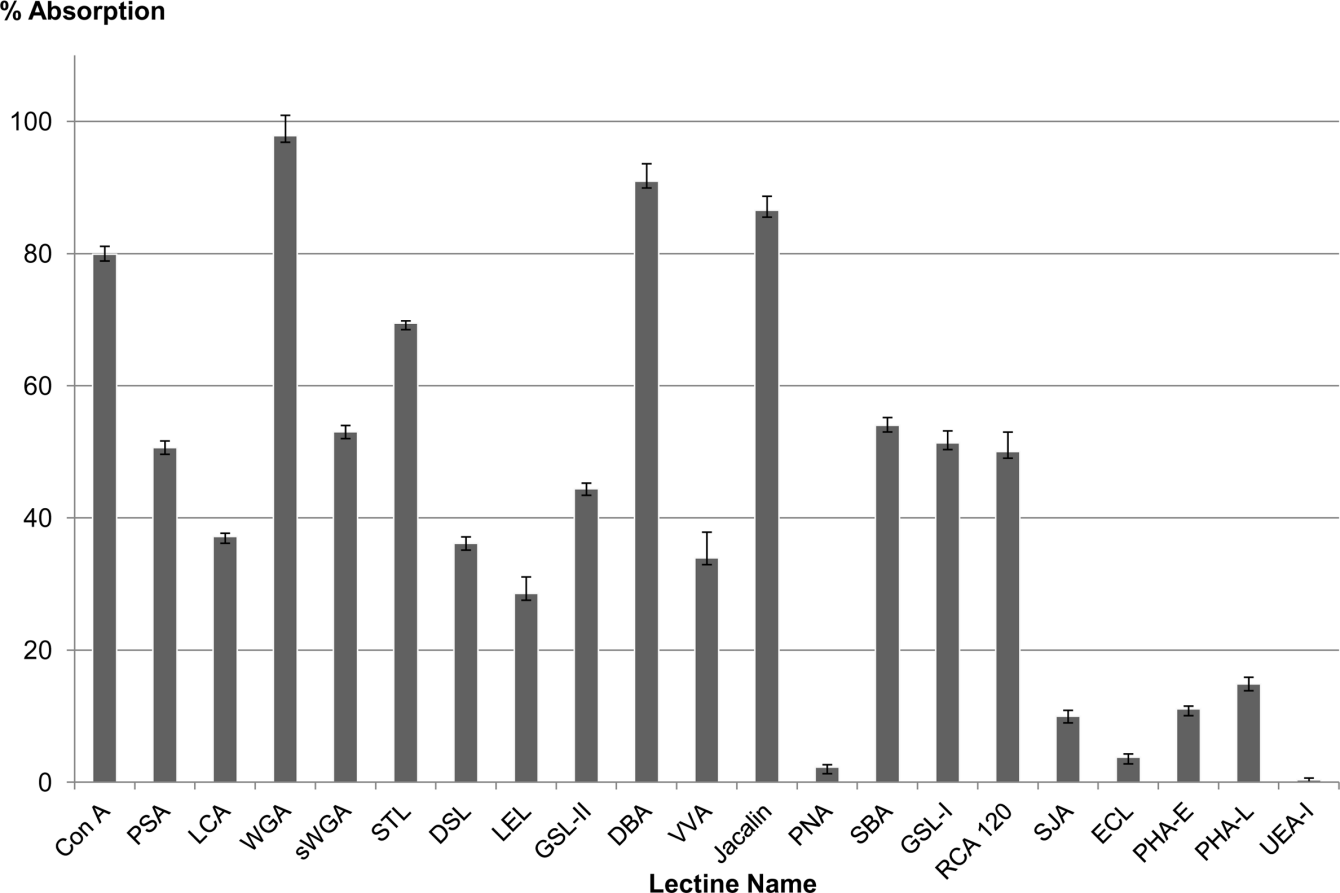
Enzyme-Linked Lectin Assay (ELLA) with a set of 21 lectins against the ASM organic matrix of the *Dreissena polymorpha* shell. The results were normalized to the highest response (Wheat germ agglutinin, WGA) which corresponding to 100%.

The results of the monosaccharide analysis are shown on Table 5 and the results of the lectin profiling of the ASM, on Fig 5. The monosaccharide content reveals a peculiar composition, where the proportions of each sugar residue is similar in the AIM and in the ASM. In particular, glucosamine represents the main sugar in both fractions, almost 58% in the AIM, and 38% in the ASM. Three other sugars represent high to moderate percentages in the two fractions: galactosamine, glucose and galactose (between 8.2 and 25.8%). Fucose, mannose and xylose are minor components (between 0.6 and 3.8%). Glucuronic and galacturonic acids, as well as arabinose and rhamnose are absent from both samples. In total, the glycosylation of the ASM and of the AIM is rather low, representing slightly more than 3% of both matrices.

**Table 5:** Monosaccharide analysis of the AIM and ASM fractions of *Dreissena polymorpha* shell.

Monosaccharides	AIM		ASM	
	ng μg^-1^	%	ng μg^-1^	%
fucose	0.5	1.6	1.2	3.5
mannose	0.6	1.9	1.3	3.8
xylose	0.2	0.6	0.4	1.2
galactose	3.4	10.7	2.8	8.2
glucose	4.3	13.6	8.75	25.8
galactosamine	4.5	14.2	6.4	18.9
glucosamine	18.2	57.4	13.1	38.6
galacturonic acid	ND	-	ND	-
glucuronic acid	ND	-	ND	-
arabinose	ND	-	ND	-
rhamnose	ND	-	ND	-
Total	31.7	100	33.95	100
Total % carbohydrates	3.2%		3.4%	

ND: not detected

A set of 21 lectins was tested to perform structural characterization of saccharide moieties of the shell ASM via an enzyme-linked lectin assay, *i*.*e*., ELLA. For technical reasons, because of its high insolubility, AIM could not be tested in solution in microplates. LS-AIM was not tested either because of its reduced binding ability to the microplate polymer. In the histogram of Fig 5, the lectins were clustered according to their sugar or motifs affinity. ConA, PAS and LCA, which bind D-mannose, D-glucose and N-acetyl-glucosamine, give a high response (from 38 to 80%). Positive signals are also obtained with the set of lectins that bind to chitin, to N-acetyl-glucosamine and to N-acetyl-lactosamine, namely WGA, sWGA, STL, DSL and LEL. Note that the highest response (100%) is given by the wheat germ agglutinin (WGA), and the lowest, by LEL (about 30%). GSL-II, which specifically binds to terminal, non-reducing α or β linked N-acetyl-D-glucosamine, gives a signal above 40%. DBA and VVA bind to N-acetyl-galactosamine: although both reactive with terminal residues, they give contrasting responses: while the second highest response (about 90%) is provided by DBA, that of VVA is moderate: (>40%). This different response may be related to the polypeptide chain neighbouring the sugar residue (see S1 Table). Another set of lectins, which preferentially bind galactose and N-acetyl-galactosamine, give contrasted responses: jacalin, SBA, GSL-I and RCA _120_ give values ranging from 50 to 80%, while no binding is achieved with PNA, and a weak one with SJA. This contrasted situation may result from the restricted specificity of SJA, and from the presence of sialic acids in the chain that may inhibit the binding of PNA. This may also occur with ECL, another lectin, with binding ability to N-acetyl-lactosamine, to lactose, to N-acetyl-D-galactosamine and to galactose, dependent on the presence of sialic acid residues. In the present case, hardly any binding is recorded with ECL lectin. Moderate binding is obtained with PHA-E and PHA-L, two lectins that bind mannose, N-acetyl-glucosamine, disaccharides containing N-acetyl-glucosamine, galactose or mannose, and a trisaccharide containing galactose, N-acetyl-glucosamine and mannose, these saccharide residues being preferentially N-linked via asparagine. At last, UEA-I, a lectin that is specific for terminal α-L fucose residues, does not give any reactivity.

### *In vitro* functional assays: inhibition and crystallization

**Fig 6:**
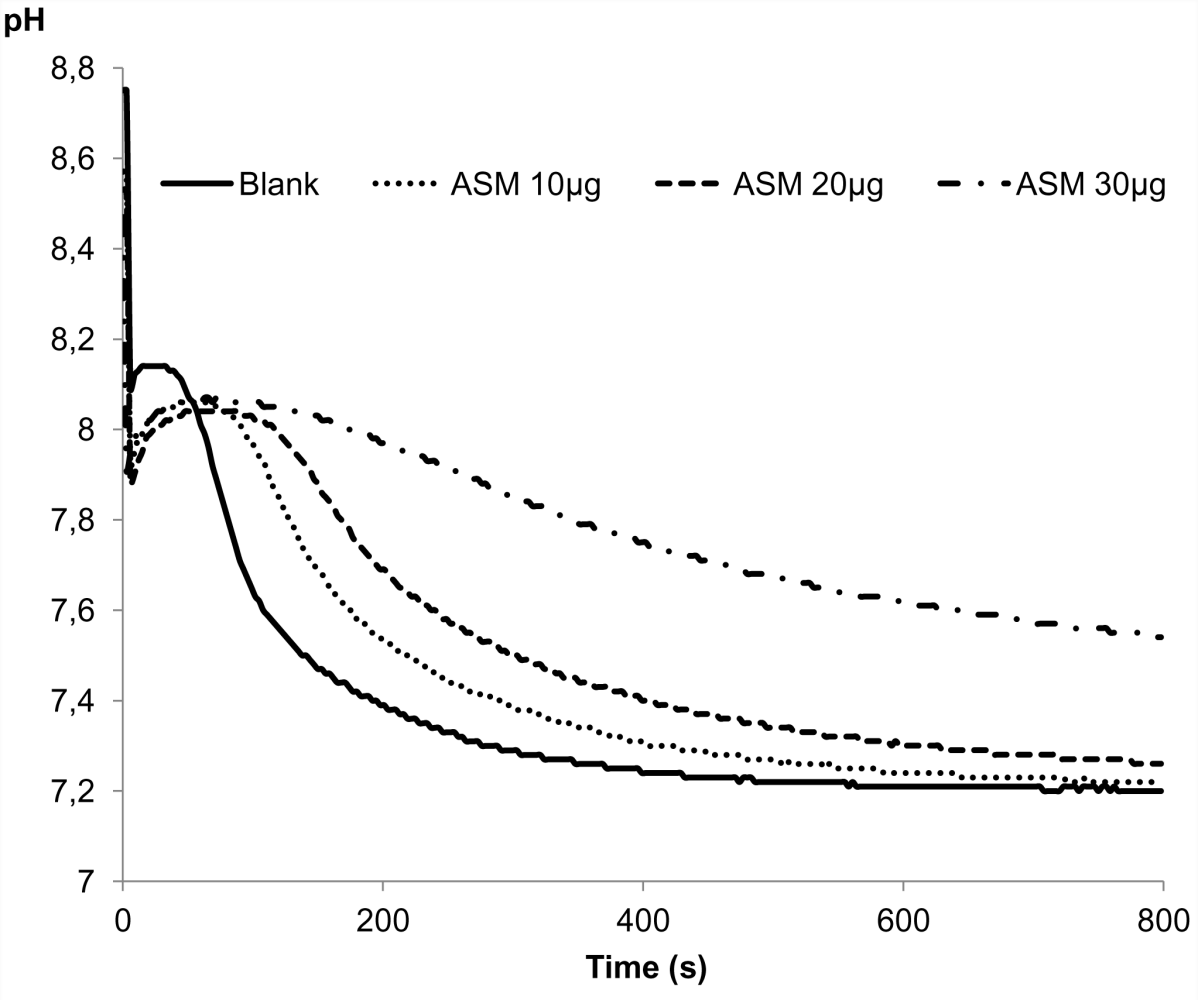
Capacity of different amounts of *D*. *polymorpha* ASM to inhibit the *in vitro* precipitation of calcium carbonate. Different quantity of ASM are tested: ••• 10, **---** 20, **-•-** 30 μg ASM.

**Fig 7:**
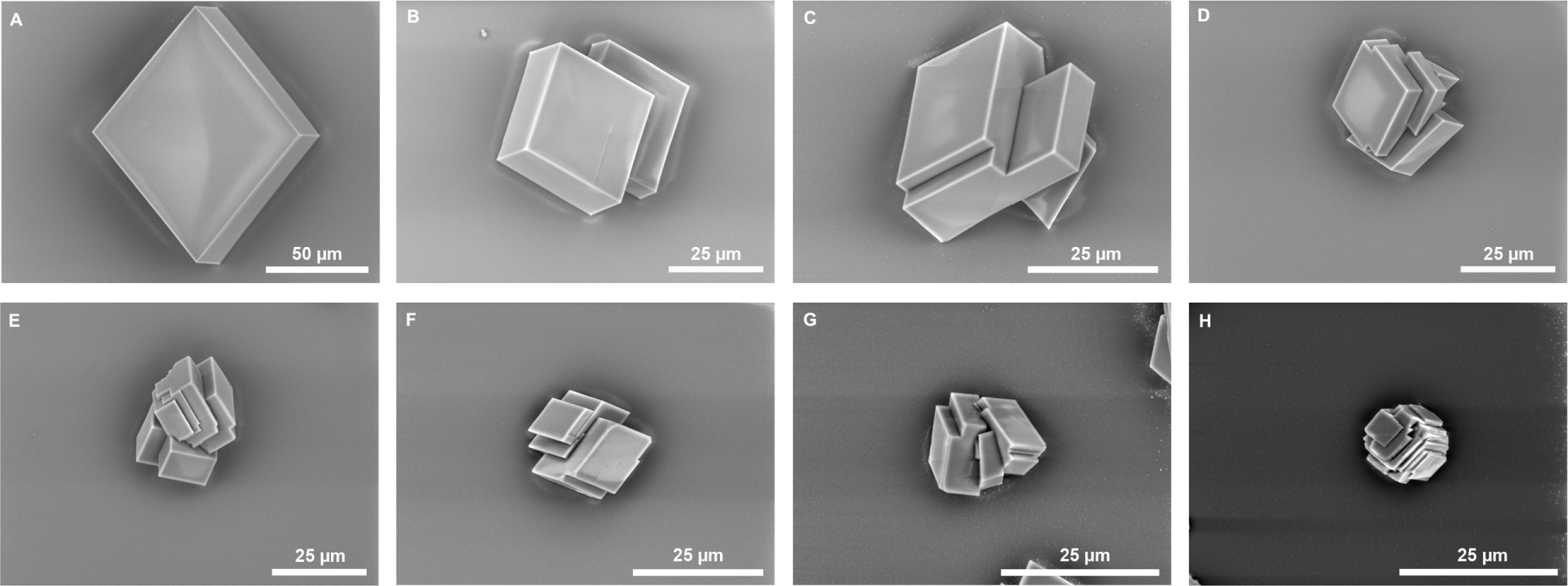
Influence of *D*. *polymorpha* on *in vitro* crystallization. Different concentrations of *D*. *polymorpha* ASM are tested for their activity on the growth of calcium carbonate crystals. A) Blank control: no protein was added; B) 0.39 μg/mL; C) 0.78 μg/mL; D) 1.56 μg/mL; E) 3.13 μg/mL; F) 6.25 μg/mL; G) 12.5 μg/mL; H) 25 μg/mL.

The results of inhibition and crystallization assays, performed solely on ASM because of its solubility, are shown on Figs 6 and 7, respectively. The capability of the ASM to inhibit the *in vitro* precipitation of calcium carbonate was checked by testing a range of ASM concentrations, from 10 to 30 μg. The matrix reacts in a dose-dependent manner: at 10 μg, the pH drop is slightly delayed (about 40–50 sec.) in comparison to the blank experiment; at 20 μg of matrix, the delay is extended to 100 sec. At 30 μg of matrix, the inhibition is stronger, although not complete since no plateau is obtained. These results suggest that the inhibitory capacity of the ASM of the shell of *D*. *polymorpha* is moderate.

The results of the crystallization assay (Fig 7) are consistent with the inhibition experiments at low concentrations of ASM (0.39 to 1.56 μg/mL), the effects of the matrix on the crystal morphology are reduced: crystals exhibit the habitus of calcite, and include the formation, and simple polycrystalline aggregates are formed (Fig 7C and 7D). At higher concentrations (3.13 to 12.5 μg/mL), more complex polycrystalline aggregates are formed, but the evolution of the morphologies in this concentration range is not marked. At 12.5 μg/mL, there are slightly more rounded angles and corners of the crystals (Fig 7G). At 25 μg/mL, the effect of the ASM on the crystal morphology is more visible: formation of polycrystalline aggregates, rounded corners, and decrease in the size of the crystals. In conclusion, the ASM exerts an effect on the morphologies of the crystals; however, this effect is moderate, in comparison to the matrix of other bivalve shells.

### Mono- and bi-dimensional gel electrophoresis

**Fig 8:**
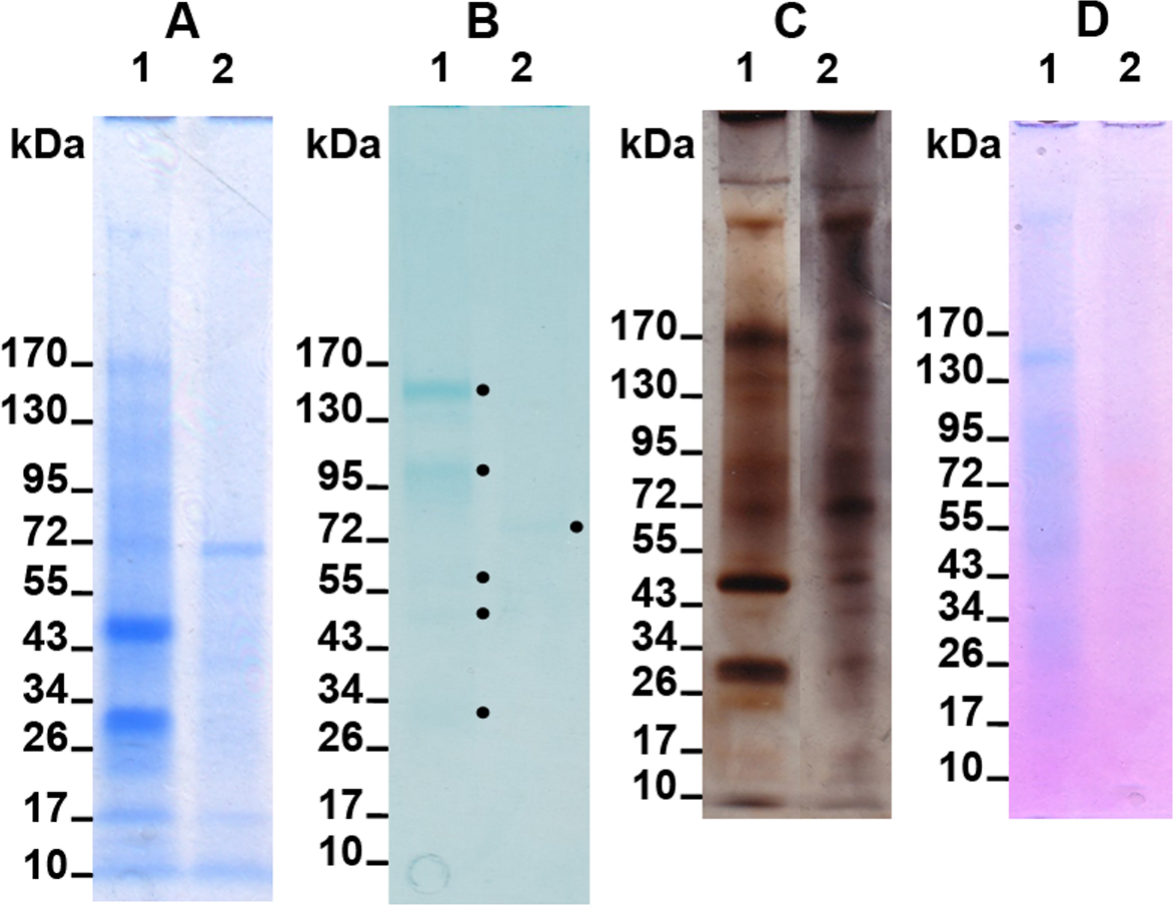
One-dimensional electrophoretic pattern of the ASM (lane 1) and of the LS-AIM (lane 2) on 4–20% SDS-PAGE gel with different staining. A) Coomassie Blue stained gel (CBB). B) Alcian blue at low pH stained gel. Black points (•) highlight five discrete bands in the ASM and one in the LS-AIM (around 80 kDa). C) Silver stained gel. D) ‘Stains-all’ stained gel.

The results of the 1D electrophoretic pattern of the ASM and of the LS-AIM are shown on Fig 8. Both matrices comprise a number of discrete molecular weight bands and of polydisperse components that represent the smear, particularly visible in the silver stained gel (Fig 8C). In the Coomassie stained gel (Fig 8A), about a dozen bands can be enumerated in the ASM (lane 1), most of which are concentrated in the 10–170 kDa range. Note that two extremely high molecular weight components are detected in the upper part of the gel. The LS-AIM fraction (Fig 8A, lane 2) exhibits discrete fractions, although less numerous than the ASM, in the Coomassie stained gel. Staining with silver nitrate allows visualizing additional bands in both fractions, in particular in the upper part of the gel for ASM, and in the whole profile, for LS-AIM. Alcian blue at low pH stains (Fig 8B) mainly five discrete bands in the ASM (in particular one around 150 kDa) and one in the LS-AIM (around 80 kDa) that are not easily visualized neither with Coomassie nor with silver staining. ‘Stains-all’ underlines a blue smear of the ASM as well as few discrete bands, especially the one at 150 kDa and the other at high molecular weight (Fig 8D). Note that LS-AIM stains mainly pink with ‘Stains-all’. In brief, whatever the gel staining treatment used the electrophoretic profiles of the ASM and of the LS-AIM are different, suggesting that the two fractions are not superimposable, although they contain discrete bands that migrate at the same distance.

**Fig 9:**
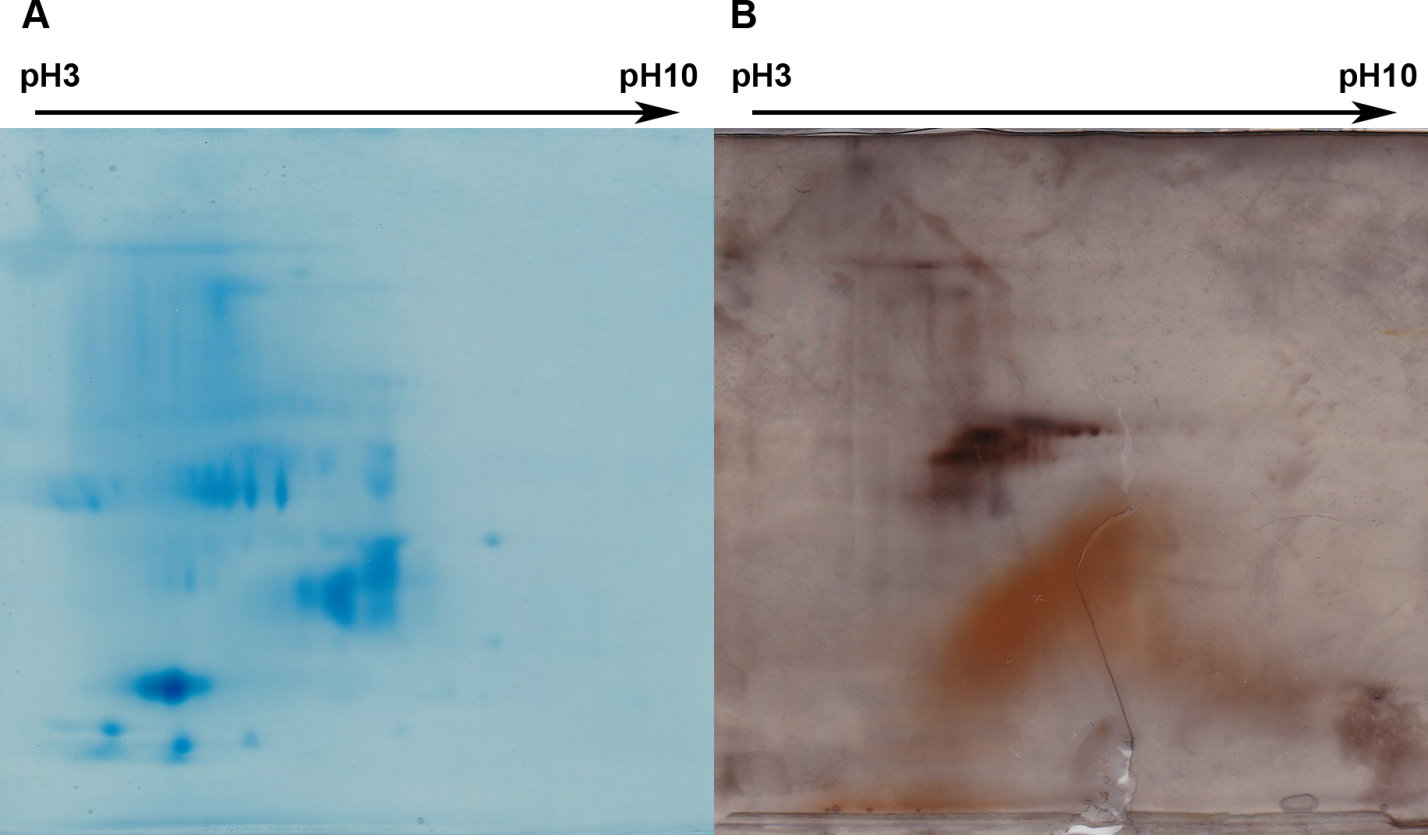
Two-dimensional electrophoretic patterns of the ASM (A) and of the AIM (B). Protein samples were loaded on a 7 cm linear pH 3–10 IPG strip and second dimension was performed with a precast 4–20% SDS-PAGE gel. ASM gel was stained with Coomassie Blue and AIM one with silver nitrate.

The 2D gel patterns are shown on Fig 9: Fig 9A corresponds to the 2D fractionation of the ASM and its subsequent staining with Coomassie, while Fig 9B, to that of the AIM stained with silver nitrate. For the ASM, one can distinguish several tens of spots and smearing materials; interestingly, most of the spots are localized below pI 7, with a few exceptions that are well marked. In the middle acidic part of the gel, a series of spots of similar apparent molecular weights are visible, suggesting different post-translational modifications, such as phosphorylations. For the AIM, less spots (than in the ASM) can be enumerated and most of them are concentrated in the acidic and middle part of the gel. A large area of smearing macromolecules is visible below the aforementioned cloud of spots.

### MS-MS analysis

The results of the MS-MS analysis are shown on Table 6. The search was performed on ‘Other Metazoa’ and ‘Other Eukaryota’, on a fusion database, the content of which is described in ‘Material and methods’ § ‘MS/MS’, and finally, on the databases of the genus *Crassostrea* and of *Paracentrotus lividus*. A limited number of about forty hits were obtained with the different databases. For ASM analysis, the in-gel digestion generated more hits than the in-solution digestion. This indicates that the purification of the matrix *via* in-gel short migration could improve the protein MS/MS identification. Nevertheless, the different digestion modalities generate not the same list of peptide sequences. Among the monospecific/monogeneric databases, that of *Pecten maximus* has identified more proteins, while no hit was found with the database of *Laternula elliptica*. The search against ‘Other Metazoa’ and ‘Other Eukaryota’ generated 18 hits, among which four correspond to biomineralizing organisms (*Chionoecetes opilio*, the snow crab; *Emiliania huxleyi*, the coccolithophore alga, *Strongylocentrotus franciscanus*, the red sea urchin and *Thalassiosira oceanica*, a diatom, *i*.*e*., a silica-forming single-cell alga). Few hits correspond to proteins of the ECM (extracellular matrix), in particular, alpha collagen, fibronectin, cathepsin and matrix metalloproteases (MMPs), while an additional hit is obtained with a protein associated to calcium, a hypothetical EF-hand calcium-binding protein of the oomycete *S*. *diclina*. One has to note also the presence of proteins that are connected to sugar metabolism (xylulose-kinase-like, galactosylceramide sulfotransferase-like, polypeptide N-acetyl-galactosaminyl-transferase 5, RIM ABC transporter) and of ubiquitin. The presence of cytoplasmic actin has been detected.

**Table 6:** Proteins identified by searching against the *Crassostrea* sp., *Paracentrotus lividus*, FUSION homemade, NCBI ‘Other Metazoa’ and ‘Other Eukaryota’ databases.

	Accesion number ^a^	Species ^b^	Protein ^c^	MASCOT score (peptides) ^d^	MW(Da) ^e^	Peptide sequence ^f^	Homology ^g^	Function Domain ^h^
**AIM Gel**	gi|33089908	*Chlamydaster sterni*	Actin	51(2)	29063	R.GYSFTTTAER.E	* *	
		* *				K.EITALAPPTMK.I	* *	* *
	gi|159507454	*Crassostrea* sp.	beta-Actin	63(3)	41771	K.AGFAGDDAPR.A		
						R.GYSFTTTAER.E		
						K.DSYVGDEAQSK.R		
	AAC32224.1	*Dreissena polymorpha*	Cytoplasmic actin	44(3)	41826	R.GYSFTTTAER.E		
						K.EITALAPPTMK.I		
						K.DSYVGDEAQSK.R		
	Singlet5583 888:2016	*Paracentrotus lividus*		88(4)		K.AGFAGDDAPR.A	Actin cytoplasmic [*Pisaster_ochraceus*] sp|P12716|	
						R.GYSFTTTAER.E		
						K.EITALAPPTMK.I		
						K.DSYVGDEAQSK.R		
	Contig10489	*Pecten maximus*		59(3)	53468	K.AGFAGDDAPR.A	Actin [*Azumapecten farreri*] gi|32816054|gb|AAP88387.1|	
	gi|113226	*Strongylocentrotus franciscanus*	Actin-15A	58(4)	41800	K.AGFAGDDAPR.A	* *	
		* *				R.GYSFTTTAER.E	* *	* *
						K.EITALAPPTMK.I	* *	* *
		* *				K.DSYVGDEAQSK.R	* *	* *
	gi|4102565	*Emiliania huxleyi*	Putative calcium binding protein	73(1)	36587	K.VASILSPR.K		
	gi|528078183	*Mesocricetus auratus*	Serum albumin precursor	54(1)	68181	K.LGEYGFQNALIVR.Y		
	gi|514701415	*Salpingoeca rosetta*	RIM ABC transporter	52(1)	270155	R.GLTLSVPR.G		Export of drugs and carbohydrates
	gi|498938245	*Ceratitis capitata*	Uncharacterized protein LOC101463398	58(1)	154190	K.YENEVAIR.G	A disintegrin and metalloproteinase with thrombospondin motifs 8 [*Ceratitis capitata*] gi|577712839|	Zinc-dependent metalloprotease
	Contig13304	*Pecten maximus*		53(1)	43803	K.VGDYGSVSGR.D	Cathepsin Z [*Columba livia*] gi|543717043|ref|XP_005499776.1|	
**AIM Sol.**	gi|5751	*Bombyx mori*	Actin A3	57(2)	41865	K.AGFAGDDAPR.A		
						K.SYELPDGQVITIGNER.F		
	gi|159507454	*Crassostrea* sp.	beta-Actin	51(3)	41771	K.AGFAGDDAPR.A		
						R.GYSFTTTAER.E		
** **						K.SYELPDGQVITIGNER.F		
** **	AAC32224.1	*Dreissena polymorpha*	Cytoplasmic actin	57(3)	41826	K.AGFAGDDAPR.A		
						R.GYSFTTTAER.E		
		* *				K.SYELPDGQVITIGNER.F		
** **	Contig12003 164:1292	*Paracentrotus lividus*		54(3)		K.AGFAGDDAPR.A	Actin cytoplasmic [*Heliocidaris_tuberculata*] sp|P69003|	
** **						R.GYSFTTTAER.E		
** **						K.SYELPDGQVITIGNER.F		
	gi|113301	*Physarum polycephalum*	Actin, plasmodial isoform	57(2)	41773	K.AGFAGDDAPR.A		
						K.SYELPDGQVITIGNER.F		
	gi|585675263	*Saccoglossus kowalevskii*	Galactosylceramide sulfotransferase-like	61(1)	44886	R.DGDSADHR.K		
	gi|193697719	*Acyrthosiphon pisum*	6-phosphofructo-2-kinase/Fructose-2,6-biphosphatase-like	51(1)	56864	R.IGGDAELSVR.G		
	Contig8748	*Pecten maximus*		44(1)	135989	K.VSLDVMVPR.V	Alpha 1 type IV collagen [*Haliotis tuberculata*] gi|268322308|emb|CBH32885.1|	
** **	Contig34595	*Pecten maximus*		40(1)	78822	K.LCDMNYQKR.Q	Collagen alpha-5(VI) chain [*Crassostrea gigas*] gi|405975735|gb|EKC40283.1|	
	gi|762136610	*Crassostrea* sp.	Uncharacterized protein	53(1)	101740	R.AVNASGCTPLHDAVKR.G	No homology	Ankyrin repeats
	gi|530729094	*Saprolegnia diclina* VS20	Hypothetical protein SDRG_14860	51(1)	77343	K.AMGASGLQGLSR.R	No homology	EF-hand, calcium binding motif
**ASM Gel**	gi|512901956	*Bombyx mori*	Xylulose kinase-like	52(1)	56965	R.LLQASGGR.A		
	gi|499007807	*Ceratitis capitata*	Protein sevenless-like	51(1)	283776	R.TAVGTPSAPR.N		Fibronectin type 3 domain
	gi|405953411	*Crassostrea* sp.	Polypeptide N-acetylgalactosaminyltransferase 5	42(1)	28492	R.SPTMAGGLFSISR.E		O-linked glycosylation of mucins
	AM230261.1	*Dreissena polymorpha*		59(1)/52(1)	11810	K.LLMQFQQESVK.C	Metalloproteinase inhibitor 3 [*Crassostrea gigas*] gi|405966706|gb|EKC31955.1|	
	EY435048.1	*Dreissena polymorpha*		55(2)	32649	K.VLEGMSVVR.K	Hypothetical protein [*Lottia gigantea*] ref|XP_009047855.1| gb|ESP01221.1|	Cyclophilin-type peptidylprolyl cis-trans isomerases
		* *				R.IVIGLFGK.T		
	Contig815 1063:2797	*Paracentrotus lividus*		42(1)		R.SPTMAGGLFSIDK.S	Polypeptide_N-acetylgalactosaminyltransferase_1 [*Homo_sapiens*] sp|Q10472|	O-linked glycosylation of mucins
	Contig36069	*Pecten maximus*		47(1)	148374	K.SSLMNLR.Q	TNFAIP3-interacting protein 2 [*Crassostrea gigas*] gi|405973741|gb|EKC38434.1|	Polyubiquitin binding domain of NEMO and related proteins
	Contig19881	*Pecten maximus*		46(1)	61066	-.AKQLPSQK.E	Origin recognition complex subunit 1 [*Crassostrea gigas*] gi|405964738|gb|EKC30187.1|	Ubiquitin-conjugating enzyme E2, catalytic (UBCc) domain
	Contig34493	*Pecten maximus*		45(1)	72632	K.QLTVDLK.C	NFU1 iron-sulfur cluster scaffold-like protein [*Crassostrea gigas*] gi|405971248|gb|EKC36094.1|	
	gi|443717462	*Capitella teleta*	Hypothetical protein CAPTEDRAFT_158600	55(1)	25057	K.VLEGMSVVR.K	Peptidyl-prolyl cis-trans isomerase [*Capitella teleta*] sp|R7UPS3|	Cyclophilin, protein folding
**ASM Sol.**	BAGiLS_004163	*Bathymodiolus azoricus*	Ubiquitin	41(1)	19533	K.ESTLHLVLR.L		
	gi|40643022	*Crassostrea* sp.	Ribosomal protein L40, partial	41(1)	10879	K.ESTLHLVLR.L		Ubiquitin
	Contig13991 1554:1940	*Paracentrotus lividus*		41(1)		K.ESTLHLVLR.L	Ribosomal protein L40, partial [*Drosophila_melanogaster*] sp|P18101|	Ubiquitin
	Contig34594	*Pecten maximus*		41(1)	94849	K.LNSCLCPQLSR.I	Zinc finger homeobox protein 3 [*Crassostrea gigas*] gi|405959418|gb|EKC25460.1|	
	gi|566017401	*Phytophthora parasitica P1569*	Hypothetical protein F443_14276	50(1)	17370	K.NQKLLLQR.V	No homology	DDE superfamily endonuclease
	gi|397610729	*Thalassiosira oceanica*	Hypothetical protein THAOC_18587	93(1)	50510	R.ACPEGADR.A	No homology	DEAD-like helicases superfamily

AIM or ASM Gel indicate MSMS identified protein from unfractionated bulk matrices digested in-gel, AIM or ASM Sol. indicate in-solution one.

a) Accession number according to those in homemade fusion database.

b) Species name derived from homemade fusion database.

c) Protein name derived from homemade fusion database.

d) MASCOT score for the entire protein. In brackets the number of unique identified peptides by MSMS MASCOT research.

e) Molecular weight of the identified protein derived from homemade fusion database.

f) Sequence of identified peptide.

g) Protein identification using BLAST homology.

h) Function domain determined using the NCBI database.

## Discussion and Conclusions

By characterizing the shell structure, chemistry and proteome of the invasive pollutant-tolerant bivalve *Dreissena polymorpha* this study lays the first foundation for correlating the biochemical properties of the shell matrix of *D*. *polymorpha* to the level of pollution of its surrounding environment. Furthermore, this study represents an attempt to link the proteomic signature of the shell to the peculiar evolutionary history of the genus *Dreissena*.

The shell microstructural observations are in agreement with previous works of Morton (1970) [63], Taylor et al. (1973) [60] and with the more detailed study of Archambaud-Guezou (1982) [59]. These three works depict the crossed lamellar (middle layer) and complex crossed-lamellar (internal layer) textures of the shell. However, Archambaud-Guezou evidenced a thin outer layer that she described as ‘lamellaire simple’ (simple lamellar). Although we agree with the existence of a thin outer layer beneath the periostracum, we observed that its microstructural characteristics are rather different from what described. The ‘simple lamellar’ terminology does not apply to the zebra mussel in the context of this study. Our work suggests instead a fish-bone pattern organized in columns growing perpendicularly to the outer shell surface.

*Dreissena polymorpha* is an efficient filter-feeder species. It is a non-buried species that attaches to the substratum via a byssus and that actively filters water of the close surroundings. Thus, comparing the quantity of minor and trace elements in the shell with that of the water can tell us more about the concentrating effect than a comparison with minor and trace elements in the sediments. Our chemical analyses clearly show that the shell of *D*. *polymorpha* acts as a selective concentrator of some trace elements: in particular, uranium, barium and zinc. Iron, manganese, strontium and silver are also concentrated in the shell although the concentration factors are unknown due to the lack of data on the concentration of each of these elements in the surrounding water. We leave aside the high content of sodium of the shell, which might result from a contamination by our bleaching treatment with NaOCl, in spite of rinsing the shells with milli-Q water. For the other divalent cations considered above, at least four mechanisms can be inferred to explain their occurrence in the shell [64–66]: incorporation into the crystal lattice (by substitution to calcium cations), adsorption on crystal planes via electrostatic interactions, entrapment in crystal defects or complexation with the organic matrix associated to the shell. Although we cannot exclude that these four mechanisms combine to different degrees for each of the aforementioned elements, it is well-documented that one of them occurs for aragonite: this mineral tolerates the incorporation of divalent cations such as Sr, Ba and U that have a larger diameter than calcium, in its crystal lattice [67–69]. Specific and targeted studies should be performed to check the contribution of the three other mechanisms, in particular, the ability of the shell matrix to bind metal ions. To conclude on these geochemical aspects, the fact that the shell of *D*. *polymorpha* concentrates uranium efficiently is an interesting property that may be exploited for the bioremediation of this radioactive pollutant in natural environments. However, one has to keep in mind that *D*. *polymorpha* is an invasive species and using large quantities of this bivalve for depolluting rivers may be detrimental to other aquatic organisms. Another consequence of the uranium concentrating effect is that the non-controlled utilization of *D*. *polymorpha* as a daily food source for poultry should be banished [70].

The concentration of organic matrix is, at slightly above 0.07% of the weight of the shell, low in comparison to nacro-prismatic shells [33,71] but is in agreement with previous findings on crossed-lamellar shell microstructures *sensu lato* [72–73]. From Palmer [74–75], such a low organic content associated with crossed-lamellar shells would correspond to a low energetic cost of synthesis of these microstructures, explaining why they tended to supplant, in the course of evolution (Cenozoic times), the matrix-enriched microstructural types, such as the nacro-prismatic ones. Another peculiarity of the shell matrix of *D*. *polymorpha* is that its soluble/insoluble ratio is close to 1. This ratio is unusual, since the insoluble shell matrix, with very few exceptions [73], tends to be more abundant than the soluble one, regardless of the microstructure.

Our quantitative data on the saccharide moieties of the shell matrix of *D*. *polymorpha* suggest that the shell AIM and ASM matrix is poorly glycosylated with overall glycosylation rate of about 3%. This tends to show that the solubility property of the shell matrix of *D*. *polymorpha* is not controlled by its glycosylation rate, in contrast to other matrices associated with calcified tissues [49]. Another remarkable characteristic is that both fractions exhibit almost superimposable monosaccharide signatures, dominated by glucosamine, glucose and galactosamine. By contrast, other shell matrices exhibit dissimilar monosaccharide signatures of their soluble *versus* insoluble fractions [72,76]. The high amount of glucosamine suggests, but does not demonstrate, the presence of chitin, while that of galactose and of galactosamine may indicate proteoglycans. Chitin has been putatively detected in several shell matrices, although its detection is generally indirect [77–78] and needs crossed approaches [79]. Although chitin is generally thought to be predominantly associated with nacreous shell layers [80], its intimate association with crossed-lamellar and complex crossed lamellar structures is likely, but should be carefully demonstrated.

The paucity of protein hits can be explained by three combined limiting factors. The absence of a transcriptome from mantle tissues of *D*. *polymorpha* precludes any unambiguous identification. Most of the proteins sequences available for *Dreissena polymorpha* come from Expressed Sequence Tags of the foot and the gills, which are non-calcifying tissues, generating a high risk of missing important transcripts specifically expressed in the mantle. Due to the low availability of transcriptome data, the closest representatives against which homology search was performed belong to the Pteriomorphia sub-class. However, this clade diverged from Heterodonta (the sub-class which comprises *Dreissena*) deep in the Palaeozoic times [81]. This means that any homologous proteins present in the two clades may have considerably drifted in term of sequence similarity.

A remarkable finding of our MS/MS analysis is that ASM and AIM do not exhibit similar peptide/protein profiles, which strongly suggests that these two fractions are truly dissimilar, which, in other words, suggests that macromolecular components of these fractions perform different functions in biomineralization. This finding is also corroborated by the two different FT-IR spectral signatures of the ASM and of the AIM, respectively. While ASM is characterized by amide peaks of high amplitude (corresponding to protein signature), that of AIM features two peaks at 1200 and 1146 cm^-1^ that may correspond to phosphate groups. Interestingly, we observed similar FTIR fingerprints for other AIM matrices, studied in previous works and related to different marine organisms ([49] and unpublished observations). The finding that dissimilar peptide compositions occur for AIM and ASM should not be generalized to other calcifying models: we observed indeed the opposite phenomenon, *i*.*e*., overlapping peptide compositions of the ASM and AIM, when analysing by proteomics the shell matrix of *Nautilus macromphalus* and of *Unio pictorum* [82]. The reason for such contrasted situations in different mollusc models is not known, since the extraction protocol is completely standardized and reproducible.

The majority of the protein hits correspond to ‘housekeeping proteins’ (ubiquitous), involved to different degrees in biomineralization, such as cyclophilins, fibronectins, cathepsins, collagens, metalloproteases and proteins that exhibit EF-hands. Such proteins have already been identified in other calcifying mollusc models, including the giant limpet *Lottia gigantea* [22], the pearl oyster [20] and the Zhikong scallop [83]. Some protein hits are new and not found elsewhere: xylulose kinase-like, galactosylceramide sulfotransferases-like, RIM ABC transporter (involved in carbohydrate export), polypeptide N-acetylgalactosaminyltransferase (involved in O-glycosylation of mucins). Strangely, we did not detect any peptides/proteins that have a typical domain signature identified in several shell proteins, the Repetitive Low Complexity Domains (defined as RLCDs). They comprise a large set of different domains, such as acidic (D/E-rich), hydrophobic (A/G-rich), basic (R/K-rich) and S/T-rich ones [84].

Finally, besides laying the foundation for correlating the biochemical properties of the shell matrix of *D*. *polymorpha* to the level of pollution of its surrounding environment, our study has remarkable evolutionary implications. In particular, the weak similarity of our shell peptide/protein content with better-known mollusc models calls for an explanation. The mollusc shell first appeared around 440 million year ago. Recent publications tend to show that shell protein assemblages, also defined as the ‘molecular toolbox’ used for shell construction, is composed of a mixture of ancient and conserved proteins, such as carbonic anhydrase, and of proteins with a rapid rate of evolution [24,85,86]. There is the possibility that the shell of *D*. *polymorpha* is constructed from a very specific set of proteins that are not identified in other mollusc taxa. Such a finding may be compatible with the peculiar origin and evolution of the group: Dreissenids (Dreissenidae) represent a recent and small family of heterodont bivalves that comprises three extant (*Congeria*, *Dreissena* and *Mytilopsis*) genera, which originates from the Lower Eocene (> 50 my). The genus *Dreissena* appeared during the Miocene, and was apparently endemic of the Parathetys area, a large E-W shallow water sea extending from central Europe to Asia, and separated from the main Thetys Sea by the Dinarid/Anatolian massif/island. During the Late Miocene (11 my), due to Southern tectonic compressive forces related to the alpine cycle, the Parathetys, fragmented into Lake Pannon on the West (Croatia, Hungary, Romania and Serbia) and Eastern Parathetys, the remnants of which are nowadays the Black and the Caspian Seas. The isolation of Lake Pannon was accompanied by a change in the water chemistry, the water becoming brackish then slowly freshening; this corresponded to a major radiation event of the genus *Dreissena* [11–12]. Later on, *Dreissena* could colonize again freshwater environments on the East, when Lake Pannon vanished, concomitantly to the Messinian episode (5 my). Only recently (18^th^-20^th^ century), the species *Dreissena polymorpha* colonized fresh waters from all Western Europe, with the construction of waterways [13]. Such an evolutionary pattern, which resembles a case of ‘island evolution’, may have led to considerable genetic drift in the protein assemblage required for shell construction. If so, this would prove, once again, that proteins associated with shell construction, exhibit a remarkable plasticity and evolvability.

## Supporting Information

S1 Table Binding preferences and specificities of each lectin according to [36–46], EY Labs. [47] and Vector Laboratories [48]. (PDF)

S2 Table Details of MS/MS analysis. (XLSX)
