# Supplementary material for: The Shell of the Invasive Bivalve Species Dreissena polymorpha: Biochemical, Elemental and Textural Investigations
Source: PLoS One. 2016 May 23;11(5):e0154264. doi: 10.1371/journal.pone.0154264 (PMC4877012; doi:10.1371/journal.pone.0154264)
Supplement: S1 Table — (PDF) [file pone.0154264.s001.pdf]

Supplementary Table S1. Binding preferences and specificities of each lectin according to [36-46], EY Labs. [47] and Vector Laboratories [48]

Latin Name	Common Name	Abbreviation	Sugar residue and/or oligosaccharide structure binding preferences
**D-Mannose / D-Glucose / N-acetylglucosamine**			
Canavalia ensiformis	jack bean	Con A	[GlcNAc(β1–2)Man(α1–6)] [GlcNAc(β1–2)Man(α1–3)] Man(β1–4)] GlcNAc(α) > D-Man(α-) > D-Glc(α-) > N-acetyl-D-glucosamine(α-). Low affinity for tri- and tetraantennary complex N-glycoproteins. Mannose-binding lectins of N-linked glycopeptides (asparagine linked). Con A recognizes α-linked mannose present as part of a "core oligosaccharide". Binding prevented when one Man of the core is substituted at C-2 and C-4 [37].
Pisum sativum	garden pea	PSA	[GlcNAc(β1–2)Man(α1–6)] [GlcNAc(β1–2)Man(α1–3)] [Man(β1–4)Man(α-)]. *Pisum sativum* agglutinin is nearly identical in structure and carbohydrate specificity to *Lens culinaris* agglutinin. The lectin has specificity toward α-linked mannose-containing oligosaccharides. Mannose dendrimers are particularly effective ligands . Although mannose (or glucose) is the primary determinant for binding. Binding enhanced by α-fucosylation at the 6-position of the N-linked GlcNAc of glycoproteins (asparagine linked) and by the presence of LacNAc or terminal mannose. Also, many glycoproteins containing branched mannose structures do not react. Low affinity for tetra-antennary glycoproteins. Binding prevented when one Man of the core is substituted at C-2 and C-4 [37].
Lens culinaris	lentil	LCA	[GlcNAc(β1–2)Man(α1–6)] [GlcNAc(β1–2)Man(α1–3)] Man(β1–4)] GlcNAc(α) > D-Man(α-) > D-Glc(α-) > N-acetyl-D-glucosamine(α-). LCA recognizes sequences containing α-linked mannose residues but recognizes additional sugars as part of the receptor structure, giving it a narrower specificity than Con A. A fucose linked α(1,6) to the core GlcNAc of N-linked glycopeptides (asparagine linked) is an important determinant for lectin activity. This specificity is also shared by the lectin from *Pisum sativum* (PSA), but not by Con A. Subtle differences in carbohydrate affinity have been shown to exist between LCA and PSA [37].
**Chitin-binding lectins / N-acetylglucosamine / N-acetyllactosamine**			
Triticum vulgare	wheat germ	WGA	GlcNacβ(1,4) GlcNacβ(1,4) GlcNac > GlcNacβ(1,4) GlcNac > GlcNac >> sialic acid (Neu5Ac) >> GalNAc. Chitin-binding lectins. N-acetylglucosamine, with preferential binding to dimers and trimers of this sugar. WGA can bind oligosaccharides containing terminal N-acetylglucosamine or chitobiose. Bacterial cell wall peptidoglycans, chitin, cartilage glycosaminoglycans, and glycolipids can also bind WGA. Native WGA has also been reported to interact with some glycoproteins via sialic acid residues (see succinylated WGA). WGA reacts strongly with the chitobiose core of asparagine linked oligosaccharides, specifically with the Man β(1,4)GlcNAc β(1,4)GlcNAc trisaccharide. It has been suggested that WGA also has an affinity for N-acetylneuraminic acid (Neu5Ac , sialic acid), since the binding of WGA to animal cells can be decreased or eliminated by treatment of the cells with neuraminidase. The precipitation of ovine submaxillary mucin OSM by the lectin is indicative of the sialic acid specificity since OSM is devoid of GlcNAc residues. Desialylated OSM contains terminal O-linked α–GalNAc residues. WGA also recognizes this carbohydrate, but to a lesser degree than either GlcNAc or sialic acid. Several analogs of Neu5Ac are also inhibitors of WGA but N-acetylglycolylneuraminic acid (Neu5Gc) is not inhibitory. Both GlcNAc and Neu5Ac are commonly found in cellular glycoproteins and in various tissue types [37].
Triticum vulgare	wheat germ	sWGA	GlcNacβ(1,4) GlcNacβ(1,4) GlcNac > GlcNacβ(1,4) GlcNac > GlcNac >> GalNAc. Succinylated Wheat Germ agglutinin does not bind to sialic acid residues, unlike the native form, but retains its specificity toward N-acetylglucosamine. Using conjugates of the native lectin and the succinylated form can provide a system to distinguish between sialylated glycoconjugates and those containing only N-acetylglucosamine structures. Native WGA can be modified by succinylation to yield a lectin which no longer reacts with sialic acid but which still retains its other carbohydrate binding properties [36].
Solanum tuberosum	potato	STL	[GlcNAcβ(1,4)]3 GlcNAc > [GlcNAcβ(1,4)]2 GlcNAc > GlcNAcβ(1,4) GlcNAc. Chitin-binding lectins. The lectin binds oligosaccharides containing β(1,4)-linked GlcNAc. The highest specificity is for trimer and tetramer of N-acetyl-D-glucosamine (GlcNAc), it possesses a similar specificity to the core (GlcNAc)2 of N-linked glycoproteins. STL also reacts with poly N-acetyllactosamine structures from N-linked oligosaccharides, albeit rather weakly. Many lectins do not react with sulfated sugars, however, STL does bind to chitin sulfates and keratan sulfates. This lectin binds oligomers of N-acetylglucosamine and some bacterial cell wall oligosaccharides containing N-acetylglucosamine and N-acetylmuramic acid. STL binds a wider set of oligosaccharide sequences than LEL [42, 45].
Datura stramonium	jimson weed	DSL	Chitotriose > Chitobiose > N-acetyl-D-glucosamine. The carbohydrate binding site recognizes (β-1,4) linked N-acetylglucosamine oligomers, preferring chitobiose or chitotriose over a single N-acetylglucosamine residue. Chitin-binding lectins. DSL also binds well to N-acetyllactosamine and oligomers containing repeating N-acetyllactosamine sequences. It is distinct among poly-N-acetyllactosamine-binding lectins in that it apparently does not require the presence of a GlcNAcβ(1-6)-linkage for interaction. A branched pentasaccharide including two N-acetyllactosamine disaccharides linked to mannose (β-1,6) and (β-1,2) was reported to be the most potent inhibitor of agglutination. Repeating units of GlcNAc and muramic acid, as found in the cell walls of *Micrococcus luteus* , are inhibitors of the lectin. This lectin binds well in the acidic pH range but its affinity decreases above pH 8.0 [42].
Lycopersicon esculentum	tomato	LEL	Chitin-binding lectins.N-acetyl-D-glucosamineb(1,4)N-acetyl-D- glucosamine oligomers up to 4 carbohydrate units. The N-acetyl-D-glucosamine residues do not need to appear consecutively, a finding that has been noted for DSL, but not for WGA or STL. LEL requires 3 consecutive LacNAc residues, making it specific for poly N-acetyllactosamine glycoproteins. Recognition of high mannose-type N glycans by LEL. Repeating units of GlcNAc and muramic acid, as found in the cell walls of *Micrococcus luteus* , are strong inhibitors of the lectin [45].
**N-acetylglucosamine**			

Griffonia simplicifolia	formerly Bandeiraea simplicifolia	GSL-II	N-acetyl-D-glucosamine. GSL-II is specific for terminal, non-reducing α- or β-linked N-acetylDglucosamine. GSL-II is the only lectin isolated that is specific for only a terminal GlcNAc residue. The subterminal saccharide does play an important role in lectin binding. GlcNAc linked β(1,3) or α(1,6) to galactose is a poor inhibitor of the lectin while GlcNAc linked α(1,3) to galactose or glucose is a potent inhibitor.
**N-acetylgalactosamine**			
Dolichos biflorus	horse gram	DBA	Terminal α-N-acetyl-D-galactosamine. α-linked N-acetylgalactosamine
Vicia villosa	hairy vetch	VVA	N-acetyl-D-galactosamine. VVA recognizes preferentially α- or β-linked terminal N-acetylgalactosamine, especially a single α-N-acetylgalactosamine residue linked to serine or threonine in a polypeptide (the Tn antigen). Evidence suggests that this lectin also may require specific amino acid sequences at the receptor site of glycosylation. The disaccharide galactosyl (α-1,3) N-acetylgalactosamine is also a potent inhibitor of this lectin [38].
**Galactose / N-acetylgalactosamine**			
Artocarpus integrifolia	jackfruit	Jacalin	α-D-Galactose and oligosaccharides terminating with this sugar, lectin is also highly specific for the T-antigen, Gal- β(1,3)GalNAc. Jacalin is useful in the purification of O-linked glycoproteins. This lectin appears to bind only O-glycosidically linked oligosaccharides, preferring the structure galactosyl (β-1,3) N-acetylgalactosamine. This structure (the T-antigen) is the oligosaccharide to which peanut agglutinin (PNA) binds. However, unlike PNA, Jacalin will bind a mono- or disialylated form of this structure. Another difference with PNA, Jacalin apparently does not bind to galactosyl-N-acetylglucosamine [41].
Arachis hypogaea	peanut	PNA	Lactose > β-D-Galactose. The T-antigen, Gal β(1,3)GalNac, is a more potent inhibitor of lectin activity than any of the monosaccharides tested. Lactose, Gal β(1,4)Glucose, is also a strong inhibitor of the lectin, indicating that the terminal non-reducing β-galactose is of primary importance. Glucose and GalNAc by themselves are not considered inhibitors of the lectin. Peanut agglutinin required this O-linked oligosaccharide to be devoid of sialic acid, whereas Jacalin will bind to the fully sialylated disaccharide [43].
Glycine max	soybean	SBA	Terminal α- and β- N-acetyl-D-galactosamine. SBA also reacts with galactose. SBA has a slight preference for α-linked sugars and contains four complementary binding sites / protein. SBA reacts more strongly with neuraminidase treated glycoconjugates, indicating a preference for terminal carbohydrates. SBA preferentially binds to oligosaccharide structures with terminal α- or β-linked N-acetylgalactosamine, and to a lesser extent, galactose residues [37].
Griffonia simplicifolia	formerly Bandeiraea simplicifolia	GSL-I	α-D-Galactoside and α-linked galactose oligosaccharides. α-GalNAc-O-Ser/Thr. αGal, αGalNAc. GSL I is a family of glycoproteins with molecular weights of approximately 114 kDa. There are two types of subunits, termed “A” and “B”, with slightly different molecular weights. These subunits combine to form tetrameric structures, resulting in five isolectins. The “A”-rich lectin preferentially agglutinates blood group A erythrocytes and thus appears to be specific for α-N-acetylgalactosamine residues, while the “B”-rich lectin preferentially agglutinates blood group B cells and is specific for α-galactose residues [44].
Ricinus communis	castor bean	RCA_{120}	RCA_{120} exhibits a specificity for β–galactose residues, with a preference for terminal sugars. RCA_{120} reacts more strongly with branched cluster glycosides than with a monosaccharide. β(1,4)-linkage is important for binding since lactose is a potent inhibitor; Gal β(1,3)glucose is only about one-third as inhibitory as lactose. N-acetyl-D-galactosamine is a very poor inhibitor of the agglutinin [37].
Sophora japonica	Japanese pagoda tree	SJA	N-acetyl-D-galactosamine > galactose. SJA has a specificity toward carbohydrate structures terminating in N-acetylgalactosamine and galactose residues, with preferential binding to β anomers. Generally, SJA reacts stronger with GalNAc than other mono-saccharides tested. Galactose linked either β(1,3) or β(1,4) to a subterminal sugar is a more potent inhibitor than GalNAc alone . GalNAc β(1,6) galactose is 15 times more inhibitory than GalNAc alone. Specific for β-N-acetyl-d-galactosamine and β-D-galactose. Binding activity of SJA seems to be enhanced at alkaline pH values [43].
**N-acetyllactosamine / N-acetylgalactosamine**			
Erythrina cristagalli	coral tree	ECL	N-acetyllactosamine > Lactose > N-acetyl-D-galactosamine > Galactose. Their primary reactivity is with terminal LacNAc structures, Galβ1,4GlcNAc. LacNAc structure is a major component of most N-linked glycoproteins . It is also reactive, to a lesser degree, with GalNAc and weaker still with galactose. Sialylation is not tolerated. Fucose prevent lectine binding [40].
**Galactose / N-acetylglucosamine / Mannose**			
Phaseolus vulgaris	red kidney bean	PHA-E	Galβ4GlcNAcβ2Manα6; (GlcNAcβ4); (GlcNAcβ4Manα3); Manβ4. Not inhibited by simple sugars. Asn-linked oligosaccharides [39].
Phaseolus vulgaris	red kidney bean	PHA-L	Galβ4GlcNAcβ6; (GlcNAcβ2Manα3); Manα3. Not inhibited by simple sugars. Asn-linked oligosaccharides [39].
**Fucose**			
Ulex europaeus	gorse, furze	UEA-I	α-L-Fucose. Strongly with α(1,2) linked fucose residues but poorly or not at all with α(1,3) or α(1,6)-linked fucose. The H type 1 trisaccharide Fuc α(1,2)Gal β(1,3) GlcNAc and the H type 2 trisaccharide Fuc α(1,2)Gal β(1,4) GlcNAc are inhibitors of the lectin. Additionally, the H type 2 trisaccharide is approximately 900 times more inhibitory than α-L-Fucose. UEA-I is unable to bind internal fucose structures [37].
